# Radical cystectomy (bladder removal) against intravesical BCG immunotherapy for high-risk non-muscle invasive bladder cancer (BRAVO): a protocol for a randomised controlled feasibility study

**DOI:** 10.1136/bmjopen-2017-017913

**Published:** 2017-08-11

**Authors:** Jamie B Oughton, Heather Poad, Maureen Twiddy, Michelle Collinson, Victoria Hiley, Kathryn Gordon, Mark Johnson, Sunjay Jain, Aidan P Noon, Rohit Chahal, Matt Simms, Mohantha Dooldeniya, Phillip Koenig, Louise Goodwin, Julia M Brown, James W F Catto

**Affiliations:** 1 Clinical Trials Research Unit, Leeds Institute of Clinical Trials Research, University of Leeds, Leeds, UK; 2 Leeds Institute of Health Sciences, University of Leeds, Leeds, UK; 3 Freeman Hospital, Newcastle, UK; 4 St James’s University Hospital, Leeds, UK; 5 Academic Urology Unit, University of Sheffield, Sheffield, UK; 6 Bradford Teaching Hospitals NHS Foundation Trust, Bradford, UK; 7 Hull and East Yorkshire NHS Trust, Hull, UK; 8 Mid Yorkshire Hospitals NHS Trus, Wakefield, UK; 10 Airedale NHS Foundation Trust, Keighley, UK

**Keywords:** high-risk non-muscle invasive bladder cancer, BCG, radical cystectomy, feasibility study, surgical trial, RCT

## Abstract

**Introduction:**

High-risk non-muscle invasive bladder cancer (HRNMIBC) is a heterogeneous disease that can be difficult to predict. While around 25% of cancers progress to invasion and metastases, the remaining majority of tumours remain within the bladder. It is uncertain whether patients with HRNMIBC are better treated with intravesical maintenance BCG (mBCG) immunotherapy or primary radical cystectomy (RC). A definitive randomised controlled trial (RCT) is needed to compare these two different treatments but may be difficult to recruit to and has not been attempted to date. Before undertaking such an RCT, it is important to understand whether such a comparison is possible and how best to achieve it.

**Methods and analysis:**

BRAVO is a multi-centre, parallel-group, mixed-methods, individually randomised, controlled, feasibility study for patients with HRNMIBC. Participants will be randomised to receive either mBCG immunotherapy or RC. The primary objective is to assess the feasibility and acceptability of performing the definitive phase III trial via estimation of eligibility and recruitment rates, assessing uptake of allocated treatment and compliance with mBCG, determining quality-of-life questionnaire completion rates and exploring reasons expressed by patients for declining recruitment into the study. We aim to recruit 60 participants from six centres in the UK. Surgical trials with disparate treatment options find recruitment challenging from both the patient and clinician perspective. By building on the experiences of other similar trials through implementing a comprehensive training package aimed at clinicians to address these challenges (qualitative substudy), we hope that we can demonstrate that a phase III trial is feasible.

**Ethics and dissemination:**

The study has ethical approval (16/YH/0268). Findings will be made available to patients, clinicians, the funders and the National Health Service through traditional publishing and social media.

**Trial registration number:**

ISRCTN12509361; Pre results.

**Strengths and limitations of this study**

- This is an important comparison that has not been attempted before.
- This study will not determine which intervention is the superior treatment; a definitive phase III trial will still be needed.
- Recruitment may be challenging and may not be possible through traditional care pathways.

## Introduction

### Context

Bladder cancer (BC) is a common disease that is one of the most expensive malignancies to manage.^1^ Around 25% of patients present with poorly differentiated, low-stage tumours, termed ‘high-risk non-muscle invasive bladder cancer’ (HRNMIBC; including tumours with carcinoma in situ, invasion into the lamina propria and intraepithelial spread into the prostatic urethra). The two main treatment options for HRNMIBC are intravesical immunotherapy (using a maintenance regime of intravesical maintenance BCG (mBCG)) and radical cystectomy (RC). The former aims to induce an immune response against the tumour and may reduce the risk of progression to muscle invasion.^2^ While mBCG avoids bladder removal, it leaves patients at risk of local progression and may impact on quality of life (QoL) through local symptoms and anxiety. RC removes the risk of local disease progression and may have the best oncological outcomes but could be overtreatment for non-progressing tumours. Many patients develop short-term postoperative complications after RC, and others have a reduction in QoL following surgery. To date, RC and mBCG have not been directly compared. Their comparative risks and benefits are unknown, hampering decision making, clinical care and exposing patients to both overtreatment and undertreatment.

### Current knowledge

The natural history of HRNMIBC is unpredictable. Rates of progression to muscle invasion and metastases vary between 25% and 75%,^3^ and long-term outcomes suggest around 20%–25% of patients with HRNMIBC may die from BC.^4 5^ mBCG avoids bladder removal, and meta-analyses report potential reductions in progression by 5% at 2.5 years^6^. However, mBCG can be poorly tolerated, its impact on progression is debated^2^ and there are manufacturing problems.^7^ mBCG involves 27 intravesical instillations and 10 cystoscopies over 3 years. Many (74%) patients report local and systemic toxicity,^8 9^ so only 30% of patients complete mBCG.^9 10^ Furthermore, there are few data to support that mBCG with bladder preservation preserves a good QoL. With regards to oncological outcomes, reports of BCs failing mBCG find upstaging to invasion in 27%–63% of tumours and the cancer-specific survival is worse than for BC with de novo muscle invasion (eg, 37% vs 67%/3 years).^11–15^ RC includes removal of the bladder and adjacent organs and reconstruction of urinary drainage. Many patients develop short-term bowel, respiratory or cardiovascular problems, including up to 20% require intervention.^16^ Prospective studies report that recovery of QoL following RC takes 6 months or longer to recover to preoperative levels.^17^ Recurrence-free survival rates following primary RC for HRNMIBC cancers appear superior to those from mBCG (eg, 79%/10 years).^18^

### Surgical RCTs

As contemporary data challenge the role of mBCG^2^ and lessons have been learnt from large surgical randomised controlled trials (RCTs),^19^ we believe it is time to compare mBCG with RC. This is an important comparison, and this opportunity may be lost as RC for HRNMIBC becomes more popular.^20^ Importantly, the 2015 NICE BC guidelines selected this comparison as one of the highest-ranked research priorities in the disease.^21^ The BRAVO study aims to compare surgical and non-surgical treatments. Trials of similarly disparate treatments in BC have previously failed to recruit (eg, CRUK-SPARE trial).^22 23^ Here we propose the preliminary work necessary to understand if we can undertake a large RCT of mBCG versus RC. Anticipated barriers to recruitment include patient and clinician preferences, BC treatment pathways, a lack of high-quality information^24 25^ and the need for staff training in equipoise and communicating RCT methods.^26^ To address these issues, we will develop a tailored staff training package to facilitate informed decision making about participation and to better understand RCT methodology. The development work will be informed by existing knowledge^24 27 28^ and context-specific evidence derived from interviews with patients and healthcare staff exploring: (a) treatment perceptions, (b) patient pathways to treatment, (c) barriers to participation and (d) training needs of site staff. This qualitative work to develop and deliver the training package is described in a separate protocol (see online supplementary file 1). We will then undertake a feasibility study to assess whether recruitment could be achieved in a definitive trial, embedding a qualitative component to establish patient experience.

10.1136/bmjopen-2017-017913.supp1 Supplementary file 1

### Study aims

Our aims are to assess whether a larger phase III RCT is possible and to acquire sufficient 129 data to aid planning such a trial. Primary outcomes are:

- To assess the number of patients screened and identified as eligible within these six centres.
- To assess recruitment rates (number of patients randomised per month).

Secondary outcomes are:

- To assess acceptance of allocated treatment.
- To assess the rate of compliance with mBCG at 12 months after randomisation and collect reasons for non-compliance.
- To assess the feasibility and optimal frequency of collecting QoL data in patients treated for HRNMIBC.
- To obtain preliminary data on the QoL data of patients treated for HRNMIBC.
- To explore the reasons expressed by patients for declining recruitment into the study.

## Methods and design

### Trial design

**Figure 1:**
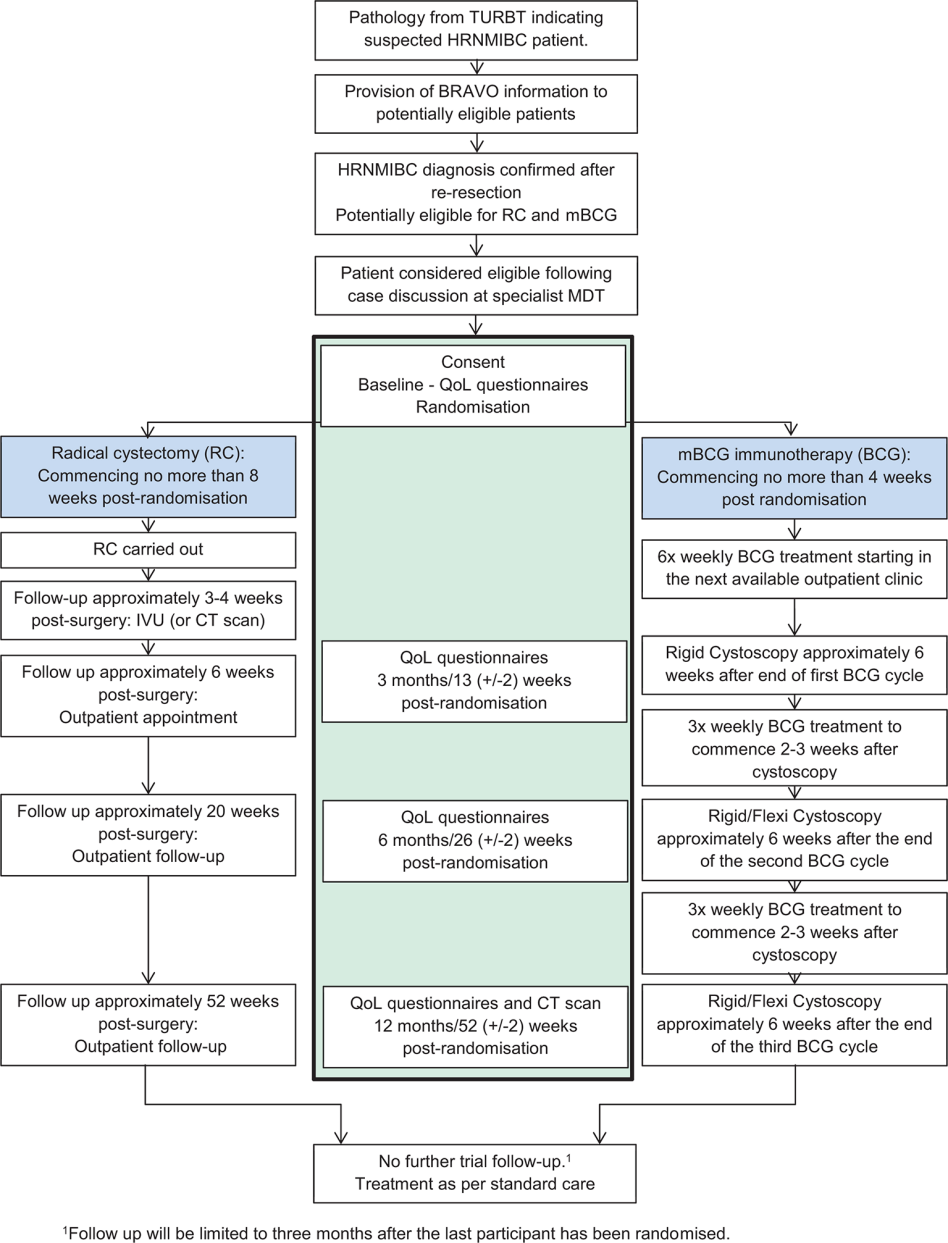
Study flow diagram. HRNMIBC, high-risk non-muscle invasive bladder cancer; IVU, intravenous urogram; MDT, multidisciplinary team; QoL, quality of life; TURBT, transurethral resection of bladder tumour.

BRAVO is a multi-centre, parallel-group, mixed-methods, individually randomised, controlled feasibility study in patients with HRNMIBC suitable for treatment by either mBCG or RC. Eligible, consenting patients will be randomised (1:1) to receive either mBCG or RC (figure 1). Due to the different treatment modalities in the two arms, it is not feasible to blind patients or clinicians to treatment allocation. Patient reported outcome data will be collected at 3, 6 and 12 months postrandomisation in clinic or by postal questionnaire if the patient is not due to attend a clinic visit.

### Trial population

We aim to recruit 60 patients from six UK cancer centres and their associated district general hospitals. The inclusion criteria are:

1. Male or female aged ≥18 years old.
2. Patients with a new diagnosis of high-risk (high-grade^29^ or grade 3^30^ non-muscle invasive urothelial carcinoma (staged as pTa, pTis or pT1). Patients with previous low-grade non-muscle invasive bladder cancer (NMIBC) are eligible.
3. The tumour is either solely urothelial cell carcinoma (UCC) or has UCC as the majority histological component.
4. In addition to the HRNMIBC bladder tumour, there needs to be one or more risk factor from:
  - presence of pTis in the bladder
  - presence of pTis in the prostatic urethra
  - lymphovascular invasion
  - vascular invasion
  - residual grade 3/high-grade UCC on re-resection (or initial transurethral resection of bladder tumour (TURBT) if no re-resection)
  - multifocal disease (>3 tumours at initial resection)
  - young age (<65 years old)
  - initial tumour size >3 cm (or >5 g in histology specimen)
  - pT1 stage.
5. Either re-resection of the bladder (following the initial diagnostic TURBT) within the 3 months prior to randomisation confirming the absence of muscle invasion
  OR
  - the initial diagnostic TURBT biopsy contains muscle, AND
  - the radiological and pathological stage assessment are in agreement regarding stage and absence of muscle invasion, AND
  - a re-resection is not appropriate in the opinion of the treating clinician AND
  - the initial TURBT is within 3 months prior to randomisation.
6. CT or cross-sectional imaging of the abdomen and pelvis within the year prior to starting treatment.
7. Imaging of the lungs and thorax within 3 months prior to randomisation.
8. Suitable and fit for both mBCG and RC as determined by the treating clinician.
9. Central multidisciplinary team (MDT) pathological review agrees with diagnosis.
10. If female, must be (as documented in patient notes):
  - postmenopausal (no menses for 12 months without an alternative medical cause) or
  - surgically sterile (hysterectomy, bilateral salpingectomy or bilateral oophorectomy) or
  - using acceptable contraception (which must be continued for 7 days after the last dose of BCG or until RC is carried out). Women of childbearing potential must undergo a pregnancy test before randomisation.
  - not breast feeding.

The exclusion criteria are:

1. solely non-urothelial or any variant urothelial pathology
2. unable or not willing to give informed consent
3. previous high-risk (high grade or grade 3) non muscle invasive (NMI) or invasive BC
4. any previous treatment with intravesical BCG
5. any previous treatment with pelvic radiotherapy
6. any other malignancy (excluding non-melanomatous skin cancer, low-risk prostate cancer and prior low-risk BC).

Eligibility waivers are not permitted.

Prior to entry, patients must be accurately staged (eg, cross-sectional imaging (eg, CT) of the abdomen, pelvis and thorax, or bone scan if indicated, within 3 months prior to randomisation) and judged to be eligible for both treatments (anaesthetic evaluation in those with borderline fitness for RC). After trial entry, women of childbearing age must be proven to be not pregnant (pregnancy test).

### Sample size

The sample size for this feasibility study has been set to give confidence that the recruitment target for the main trial can be met. A formal power calculation is not appropriate as effectiveness is not being evaluated. It is estimated that, per year, over the six centres, there will be approximately 1000 new diagnoses of NMIBC, where 20% are likely to be eligible (200 patients).^31^ We would need to show that we are able to randomise approximately 25% of all eligible patients to be confident that the recruitment target for the main trial would be met within 3 years, with an additional nine centres. We therefore plan to recruit 60 patients over an 18-month period in the feasibility study. For the phase III trial, we anticipate either a single primary endpoint (cancer-specific survival) or coprimary endpoints (cancer-specific survival and averaged quality-adjusted life years (QALYs)). We estimate that 506 participants are required to have 80% power to show a superiority HR of 0.626 (based on an improvement in 5-year cancer-specific survival from 70% in the BCG arm to 80% in the RC arm), assuming a 3-year accrual period, 5 years of follow-up and accounting for 5% loss-to-follow-up.

### Setting

Participants will be recruited from six cancer centres (and seven neighbouring district hospitals) within Yorkshire and Northumberland. National Health Service (NHS) demographic data show that Yorkshire and Northumberland have some of the highest rates of BC incidence and some of the lowest rates of survival from this cancer.^32 33^

### Recruitment

Patients will be identified through MDT meetings and approached once they know their diagnosis of HRNMIBC. This approach may be at any hospital involved in their care and by medical or nursing staff. The team will introduce the trial when treatment options are being discussed, provide the introduction leaflet and ask permission (and contact details) for a research nurse to contact the patient with more information. The number of eligible and screened patients will be recorded. Interested participants will be invited to attend an appointment at the research site and/or receive telephone calls, to be given a full explanation of the BRAVO study. Experience in similar studies suggests that patients can be overwhelmed by information given in clinic and that telephone contact can help and provides another opportunity to support patients. Up to five attempts will be made to contact the participant by telephone, after which it will be assumed they have decided to not participate. Eligible patients can be contacted by post if the immediate care team deem this best. No contact information will be shared outside of the team directly caring for the patient unless consent has been obtained.

### Consent

Informed consent takes place in a face-to-face setting at the research site. Patients will have at least 24 hours to consider participation and will be encouraged to discuss the study with their family and other healthcare professionals. A full verbal explanation of the study, a written Patient Information Sheet (detailing rationale, design and personal implications of trial entry) and informed consent form will be provided. Participants may withdraw at any stage of the trial. Consent will be obtained prior to collection of baseline assessment data and subsequent randomisation.

### Staff training

We recognise the challenge of comparing these two treatment choices and that the patient pathway includes interaction with numerous healthcare providers. To minimise bias and to maintain equipoise, a training package will be developed from interviews with patients and clinicians and delivered to staff who are likely to care for patients before and during the study. Training will incorporate lectures and role play exercises with simulated patients. A careful explanation of the potential risks and benefits of the two treatment interventions is crucial; such risks will be clearly explained to interested patients in an unbiased and fair way, assisted by written study-specific patient information.

### Randomisation

Patients will be randomised, using a 24-hour centralised telephone or web-based randomisation system, on a 1:1 basis to receive either RC or mBCG. A computer-generated adaptive minimisation algorithm that incorporates a random element will be used to ensure that the treatment groups are balanced (stratified) for:

- age (<75,>=75)
- sex (male, female)
- recruiting cancer centre
- tumour stage (pTa/pTis, pT1)
- presence of carcinoma in situ (yes, no)
- previous low-risk BC (yes, no).

### Intervention: BCG immunotherapy

Maintenance BCG immunotherapy will be administered at either the cancer centre or district general hospital using the SWOG protocol.^10^ At least 12 months of BCG treatment are required, and 6 weeks of induction BCG will be followed by 3 doses at 4 and 10 months after diagnosis. Delays and deferrals are common and allowed within this study. BCG induction should include at least 4 (of 6) doses of BCG, and induction should be completed within 10 weeks. The presence of an invasive BC requires the cessation of mBCG and a change in treatment intent. Maintenance BCG may continue in the presence of low-risk NMI and HRNMI BC at the first cystoscopy; thereafter, these are managed as recurrences and require patient discussion. Rigid cystoscopy with bladder biopsy and bladder washings is mandated at the first check. After this, bladder surveillance is performed as per local protocol (flexible or rigid instruments). All cystoscopies will be undertaken or directly supervised (with a visual check) by a consultant urologist who manages HRNMIBC. Fluorescence or narrow band imaging may be used, as per local protocols. Histological review of the bladder biopsies and urinary cells should be performed to determine the presence or absence of BC.

Local and systemic complications are common in mBCG regimens and should be managed as per local protocol. The study will collect data on the frequency of expected BCG toxicities and whether this leads to the cessation of BCG treatment. Cystectomy may be performed within BRAVO for severe BCG-related toxicities, if these warrant such an intervention. Patients undergoing BCG treatment may stop treatment due to disease progression, disease recurrence, serious BCG intolerance or side effects or patient choice. Disease progression: patients who have confirmed progressive disease after any of the check cystoscopies (presence of pT2 tumours, cancer in lymph nodes or metastases) should stop BCG and be offered curative treatment for muscle invasive BC. Disease recurrence is defined as the presence of low-risk NMI or HRNMIBC from the second check cystoscopy onwards. Participants with recurrence should be offered the option of changing treatment, including RC or using second-line intravesical approaches.

### Intervention: RC

RC should be performed at each cancer centre by teams specialising in this service. Variations in surgical performance and practice produce wide differences in morbidity and mortality from RC.^34^ To mitigate these, surgeons within BRAVO will have individually undertaken at least 10 RCs per year for the last 2 years (or 20 in the last year), have median length of stay rates under 16 days and have a 90-day post-RC mortality rate of less than 10% (collected outcomes from the British Association of Urological Surgeons RC complex dataset).^31^ Postoperative complication rates and intraoperative and postoperative transfusion rates will also be taken into consideration. Individual surgeon data will act as surrogate measures for the entire surgical team and require accreditation from the trial management group before entry into BRAVO. Submitted data for surgical accreditation should reflect the practice to be undertaken within this study (eg, open or robotic approaches). Surgery should take place within 8 weeks of randomisation.

Cystectomy should include removal of adjacent organs. In males, this includes the prostate and seminal vesicles. In females, this should include a section of adjacent anterior vaginal wall, the uterus, cervix and fallopian tubes and, if no bladder reconstruction is planned, the urethra. Oophorectomy is optional, as per local practice and individualised for each patient. Pelvic lymphadenectomy is mandated within BRAVO. The template should at least include the regional lymph nodes up to the level of the ureteric crossing of the common iliac vessels. This includes the obturator fossa, the external iliac and internal iliac nodes. A more extended lymphadenectomy is acceptable. Excised lymphatic tissue should be submitted for histological analysis. Perioperative care is to be carried out as per enhanced recovery after surgery protocols.^35 36^

### Withdrawal of treatment

In line with usual clinical care, cessation or alteration of regimens will be at the discretion of attending clinicians or the participants. All participants who withdraw or are withdrawn from their allocated treatment will still attend for follow-up assessments and complete questionnaires unless unwilling to do so and outcomes will continue to be collected. In the event that a patient withdraws consent prior to randomisation, data collected up to the point of withdrawal will be analysed.

### Data collection

A screening form, to include demographic details and reasons for ineligibility, exclusion or refusal, will be completed for all patients considered for BRAVO. A feedback questionnaire will be used to identify patients who are willing to take part in the qualitative substudy (see online supplementary file 1). Baseline assessments prior to randomisation include QoL scores (EuroQuol-5D (EQ-5D),^37^ EORTC QLQ-C30,^38^ EORTC QLQ-BLM30) at trial entry.

Within mBCG, outcomes and compliance data will be collected at each cystoscopy. For RC, patient and operative data will be collected at the time of surgery, as per our national register,^31^ and then at each subsequent follow-up visit (3, 6 and 12 months postrandomisation). Follow-up imaging (CT scan) to assess response to treatment will be performed in both arms at 1-year postrandomisation. QoL questionnaires will be collected at 3, 6 and 12 months postrandomisation in face-to-face consultations or by telephone. These include EQ-5D,^37^ EORTC QLQ-C30^38^ and either EORTC QLQ-BLM30 (for those randomised to RC) or EORTC QLQ-NMIBC24 (for those randomised to BCG). Information will be collected on deaths, complications and toxicities (adverse events) and related and unexpected serious adverse events up to 1 year postrandomisation or 3 months after the last participant is randomised if earlier.

### Statistical analyses

A detailed statistical analysis plan will be written before any analysis is undertaken. All analyses and data summaries will be conducted on the intention-to-treat population. No formal interim analyses are planned, and final analysis will take place when all available data have been received. The analysis will focus on descriptive statistics and CI estimation. Primary analysis will include summaries of the number of patients at each stage of the recruitment pathway (screening, eligibility, consent and randomisation) and assessment of the overall monthly recruitment rate. Secondary analysis will include summaries of acceptance of randomised treatment and mBCG treatment compliance. Participant retention and self-reported QoL outcomes during follow-up, including withdrawal data (timing and reason), will also be summarised overall and by time point. Levels of missing data in QoL outcomes will be assessed. The median cancer-specific survival estimate and its corresponding 95% CI will be calculated to inform the sample size calculation of the phase III trial. As this is to aid the design of a pragmatic phase III trial, all randomised patients will be included in the calculation, regardless of treatment received. Cancer-specific survival will be calculated from the date of randomisation to the date of cancer-specific death. Participants with missing follow-up data or who are alive at the time of the analysis will be censored at the date they were last known to be alive. Overall survival, calculated from the date of randomisation to the date of death, will also be summarised as for cancer-specific survival.

The frequent collection of QoL data within this feasibility study is necessary in order to assess the burden to patients. This will be assessed by monitoring collection compliance rates and will inform the optimal frequency of data collection for the main trial. Averaged QALYs may be a coprimary endpoint for the main trial; as such, determining the optimal frequency of EQ-5D data collection within this feasibility study is crucial.

### Safety

The number of adverse events and related unexpected serious adverse events will be summarised descriptively by arm, grade and body system. The proportion of participants experiencing each toxicity will be summarised by maximum National Cancer Institute’s Common Terminology Criteria for Adverse Events grade^39^ experienced, overall and by arm. Operative RC complications will be graded using the Clavien Dindo classification.^40^

### Criteria for progression to the definitive phase III trial

The following guidelines for progression to a definitive phase III trial have been defined:

- The recruitment and follow-up rates must demonstrate that a definitive trial using similar procedures will achieve sufficient power to test the hypothesised difference between treatment arms.
- The sample size calculation for the feasibility study and proposed phase III trial are provided earlier. This assumes that 20% of all new diagnoses of NMIBC would be eligible and approximately 25% of those would be randomised. To proceed to a definitive trial, we need to show that at least 20% of eligible patients can be randomised.

### Qualitative substudy

There are two qualitative studies. The first was undertaken prior to the start of the RCT to identify a priori the barriers to recruitment from the perspectives of patients and staff to inform the development of a bespoke training package for staff^41^ (see online supplementary file 1). A second qualitative study is embedded into the RCT trial to understand patients’ views and experiences of the treatments and explore patients’ acceptability of the study and recruitment processes:

### Qualitative substudy **objectives**

1. To gauge patients’ understanding of the study and their views on the recruitment process.
2. To qualitatively explore patient’s acceptability of the study to assist in optimisation of recruitment strategies employed for the definitive trial.
3. To explore reasons for participation and non-participation of eligible patients.
4. To understand patients’ experience of the randomisation process on decision making.
5. To understand why people refuse to participate or do not take up allocated treatment.
6. Patient understanding of study materials, that is, do patients understand what will happen if they take part and do they understand what they are being randomised to.
7. Acceptability of study procedures.
8. Acceptability of randomisation.

### Qualitative substudy overview

In order to examine the views and experiences of patients with BC, we will conduct in-depth semistructured interviews with patients approached to take part in the trial. Qualitative findings will help illuminate the acceptability of trial processes and explore barriers to uptake.

Recruitment to RCTs with very different treatment arms can be difficult, and recruitment to trials involving surgery is particularly challenging.^42^ Trials present practical and methodological challenges, including difficulties in recruitment, randomisation and lack of clinical equipoise.^43^ Understanding why patients do or do not participate in trials is important, and clinical trials have recently begun to incorporate a qualitative component to address these issues. These studies have been able to successfully identify aspects of the trial design that hindered recruitment and identify possible solutions.^42 44^

### Qualitative substudy design

All eligible patients will be asked to complete a questionnaire to gauge their understanding of BRAVO and their views on the recruitment process. We will collect data from patients who decline the study, who consent but refuse allocation and who consent and accept allocation. A short questionnaire will be given to seek patient views on the recruitment process and to ask if participants would be willing to provide detailed feedback by face-to-face or telephone interview. A purposive sample of 15 patients will be selected for interview. Written consent will be taken prior to the interview and a flexible topic guide developed in conjunction with patient and public involvement (PPI) representatives, clinical colleagues and informed by the literature used to assist questioning. The topic guide will be devised to ensure that the key issues are covered but do not dictate data collection and will be flexible enough to elicit participants’ own experiences and views of the trial as well as issues unanticipated by the interview team. Interviews will be audio-recorded, transcribed and anonymised to protect confidentiality. With their consent, participants may be contacted after the interview to answer questions that may emerge during the analysis or to explore issues that emerged in the interviews in more depth.

### Qualitative substudy data analysis

Qualitative data will be analysed by the qualitative researcher. Interview transcripts will be checked for accuracy and then managed using NVivo qualitative data analysis software (QSR International, Daresbury, UK), which aids the indexing of qualitative data. Analysis will start during data collection and will inform later data collection; for example, emerging themes may identify new questions to explore in later interviews. The data will be analysed using thematic analysis^45 46^ using an inductive (bottom up) approach to identify and analyse patterns across the dataset using constant comparison methods.^47 48^ Inductive coding will follow using a line-by-line coding approach, with codes assigned to segments of data that provide insight into participants’ views of the trial. An initial coding frame will be developed from the first interviews and will be modified, if necessary, as the analysis develops. A subset of transcripts will be independently coded by another member of the team and compared with ensure consistency. Any discrepancies will be discussed with the research team and resolved to achieve coding consensus. The data will be examined for negative cases, and the reasons will be explored by comparison with the overall dataset.

### Data monitoring

Trial supervision includes a core project team, a trial management group (TMG) and an independent trial steering committee (TSC). For a feasibility study of this nature and duration, a separate data monitoring and ethics committee is not required; rather; TSC adopts a safety monitoring role and will review safety issues if this becomes necessary. Data will be monitored for quality and completeness by the Clinical and Translational Research Unit (CTRU). Missing data (except individual items collected via questionnaires) will be chased until received, confirmed as not available or the trial is at analysis. Any protocol changes will be disseminated by CTRU to the relevant parties.

### Trial organisation and administration

The trial was developed by the BRAVO TMG. The trial is funded by Yorkshire Cancer Research and is sponsored by the Sheffield Teaching Hospitals NHS Trust (Clinical Research Office, Royal Hallamshire Hospital, D Floor, Glossop Road, Sheffield), coordinated by CTRU, University of Leeds, and is registered (ISRCTN12509361). The trial will be conducted in accordance with the principles of Good Clinical Practice (GCP) in clinical trials, as applicable under UK regulations, the NHS Research Governance Framework and through adherence to CTRU standard operating procedures. CTRU/sponsor have systems in place to ensure that serious breaches of GCP or the trial protocol are identified and reported. Ethical approval has been obtained from the National Research Ethics Service Committee Yorkshire & Humber–South Yorkshire (reference no. 16/YH/0268). Any on-site source data verification carried out by CTRU is not independent from sponsor. Sheffield Teaching Hospitals NHS Trust will not be liable for negligent harm caused by the design of the trial. No additional compensation for clinical negligence will be provided for trial participants over that which is available to NHS patients. All identifiable information collected during the course of the study will be kept strictly confidential and not transferred outside of the research team. Patient name (via consent form), email address and telephone number will be collected when a patient is randomised into the study, but all other data collection forms that are transferred to or from the CTRU will be coded with a study number and will include two patient identifiers, usually the patient’s initials and date of birth. Both electronic and paper data will be held in a secure locations with restricted access.

## Discussion

The 2015 NICE BC guidelines identified the comparison between mBCG and RC as one of their highest research priorities.^21^ This reflects the importance of this question but does not address how randomisation between two very different treatment options should occur or whether such a comparison is possible. Within this feasibility study, we are attempting to understand, address and develop methodology to allow such a comparison. This will require several key issues to be addressed. First, it is clear from other surgical versus non-surgical treatment trials^49^ that the most important element for RCT recruitment is keeping equipoise when discussing the treatment options by medical and nursing staff. While previous studies used research nurses to keep equipoise, this is not viable across many centres within the current research funding climate. In an attempt to replicate this model, we ran a number of educational days to train relevant medical and nursing staff about the importance of equipoise and to discuss their beliefs about HRNMIBC. All staff had opinions about the efficacy of BCG and QoL with RC, and so it was important to discuss these in an open forum to challenge these views and use evidence to dispel prior beliefs. We proposed a six-stage consultation plan to help staff keep patients at equipoise and so facilitate trial entry and treatment acceptance.^50^ Within this feasibility study, we will determine if this approach is possible and successful. Second, UK data do not accurately identify the number of patients with HRNMIBC, what proportion of these are suitable for both RC and mBCG and how many of these would accept randomised treatment options. Within this feasibility study, we will establish accurate data about the number of eligible cases across this population and understand what proportion accepts their randomised treatment allocation. We will use these findings to power the phase III comparative study. Finally, there are very few reliable data about QoL with mBCG and none that compare this directly to RC. Within this study, we will produce these data within 60 patients (30 for each arm) and so allow this endpoint to be modelled for the larger phase III study.

## Ethics and dissemination

The study has ethical approval from Research Ethics Service Committee Yorkshire & Humber–South Yorkshire (reference no. 16/YH/0268). The results of the study will be published in peer-reviewed publications and will be presented at relevant national and international conferences. We will work with our patient panel of BC survivors to develop lay reports to disseminate research findings to patient groups and the clinical teams at participating sites.

### Availability of data

CTRU will control the final trial dataset and any requests for access will be reviewed by TMG and TSC, subject to existing contractual arrangements with the funders. The protocol, sample case report forms and participant information are available on a case-by-case basis as agreed by TMG, on request to the corresponding author.

### Trial status

The trial opened to recruitment in October 2016 using protocol version 2.0 (8 October 2016) and is due to close in March 2018.

The protocol was amended to version 3 in October 2016 to account for additional inclusion and exclusion criteria and updated surgeon accreditation criteria. The protocol was amended to version 4 in November 2016 to further update the inclusion criteria and surgeon accreditation criteria. Both amendments were reviewed and approved by the sponsor and the National Research Ethics Service Committee Yorkshire & Humber–South Yorkshire (reference no. 16/YH/0268). Protocol amendments are disseminated to relevant parties by CTRU.

## Supplementary Material

Reviewer comments

Author's manuscript
