# Supplementary material for: Radical cystectomy (bladder removal) against intravesical BCG immunotherapy for high-risk non-muscle invasive bladder cancer (BRAVO): a protocol for a randomised controlled feasibility study
Source: BMJ Open. 2017 Aug 11;7(8):e017913. doi: 10.1136/bmjopen-2017-017913 (PMC5724134; doi:10.1136/bmjopen-2017-017913)
Supplement: Supplementary file 1 [file bmjopen-2017-017913supp001.pdf]

Supplementary File 1

Qualitative Sub Study 1: Understanding patients' and health professionals' beliefs about BCG and radical cystectomy and potential barriers to recruitment.

Introduction

Lessons have been learned from previous large surgical randomised controlled trials (RCTs) (1), which suggest that when trials compare very different interventions, there are likely to be significant barriers to recruitment. A previous bladder cancer trial struggled to recruit (CRUK-SPARE trial), so this study comprises the preliminary work necessary for a feasibility RCT of mBCG versus primary radical cystectomy.

There are many barriers to recruitment and in the context of surgical trials we know that patient-factors, clinical-team factors, and information and consent related issues have all been identified as important considerations (2, 3). There will be a range of reasons for declining participation in the clinical trial including a lack of interest (4), not feeling well enough (5), fear of increased time commitments (4), and patient preferences (6). However, decisions not to participate may also be related to patients' misunderstandings regarding clinical trials (7) or how the healthcare professionals involved present the design and objectives of the study to the patient (7), and how the patient assimilates this information.

In the case of surgical trials, a need for staff training has been identified to ensure that both arms of the trial are presented in a balanced way so that patients understand the relative strengths and weaknesses of each, and there is also a recognised need for training about how to describe RCT methods (8). Radical cystectomy and BCG have been around in clinical practice for many years, so patients and health professionals may have a strong preference for either surgery or BCG and could feel that this choice is taken out of their hands by the randomization process. Understanding and addressing these issues will be crucial to the success of the feasibility trial whose aim will be to demonstrate that recruiting to a larger scale phase III trial is feasible.

It is therefore important that we have a clear understanding of patients' and health professionals' beliefs about these two treatments and ensure information presented to patients

by health professionals is done so in a way which minimises potential biases and facilitates an informed decision about participation. To address this, a tailored training package will be developed to enable staff to elicit and sensitively explore patient preferences for treatment, and facilitate an informed decision about participation. The development of the training package will be informed by existing evidence of what works (2, 9, 10) and content specific evidence derived from interviews with patients and healthcare staff to explore: a) treatment perceptions, b) barriers to participation, c) training needs of site staff.

Primary aims

- To understand patients' and professionals' beliefs about the two interventions and identify potential barriers to recruitment.
- To develop a training package for health professionals to aid informed decision making with patients

Secondary aims

- To elicit patients' beliefs and experiences of the two interventions (routes to diagnosis and beliefs about treatment options)
- To understand treatment burden and quality of life following treatment
- To elicit patient expectations of likely trial burden and barriers to participation
- To elicit patient recommendations for optimal recruitment and their views about randomisation
- To elicit health professional's beliefs about treatments, barriers to participation and perceived training needs

Outcome

Using the information gathered from the interviews and focus groups, and existing literature, develop a training package and associated materials and deliver the training package to staff to improve recruitment communication with patients.

Phase 1: ***Understanding Health Professionals views of bladder cancer treatment***

Design: Focus group study

Setting

Counselling and recruitment to the planned RCT will occur at the cancer referral centres, but patients are likely to discuss their treatment with the consultant at their local urological unit. To better understand the treatment beliefs of the health professionals (urologists, surgeons, nurses, research nurses, MDT co-ordinators and clinical nurse specialists) that patients may come in contact with, either to receive guidance on their treatment options, or to discuss the clinical trial, we approached staff from local units and referral centres. Packs were sent to the local Principal Investigator at each consenting site.

Inclusion Criteria

Staff involved in the recruitment of patients to the feasibility trial (MDT co-ordinators, surgeons, urologists, research nurses, clinical nurse specialists).

Sampling

We conducted focus groups with health professionals involved at different stages of the diagnosis pathway and trial recruitment pathway. A purposive sampling strategy was used to ensure we interviewed people involved across the diagnosis process, plus research nurses who would be involved in recruitment to the future trial. The sample included staff at local units and referral centres; nurses, (to include clincial nurse specialists and research nurses) (n=6-8), urologists and surgeons at local units and referral centres (n=6-8). We aimed to include senior and less experienced staff in each group.

Sample identification and consent process

All staff involved in the diagnosis process at each urological unit (local units and referral centres) were invited by letter to participate. An information pack (PIS, consent form, demographics form) was sent via the local Principal Investigator to their team.

Procedure

Two focus groups were undertaken (one each: nurses; clinicians); interviews (telephone or face to face) were offered to those who consented but could not attend the focus group. Focus groups were lead by an experienced qualitative researcher (MT) and supported by a second researcher. Written consent was taken at the beginning of the focus group. Discussions were informed by a topic guide which was informed by existing literature, (e.g. 9) clinical input and our PPI members, to include: beliefs about, and attitudes towards the interventions, barriers to recruitment, and training needs. The focus groups were audio-recorded with permission of the participants.

Data Analysis

Due to time and funding constraints, interviews were listened to and key sections transcribed for analysis. Personally identifiable data was removed or de-identified during transcription. The focus groups were analysed first, using an inductive, thematic coding approach. These were used to devise a coding frame for the interview transcripts. One researcher (JB) coded the remaining recordings, and a second researcher (MT) examined sections of data to check robustness of the themes.

Phase 2: Understanding patient views of bladder cancer treatments

Design: Semi-structured face-to-face interviews.

Inclusion criteria

- Aged 18 years or older
- Previous high grade (or grade 3) urothelial bladder cancer or non-muscle invasive tumour (diagnosed in previous 24 months – but not less than 4 months)
- Received either radical cystectomy or MBCG (or both)
- Able to provide written informed consent
- Able to converse in English (even if not first language)
- Currently or previously under the care of the urological units in Yorkshire and Humber.

Exclusion criteria

- Decline participation in the study
- Unable to comply with requirements of this protocol
- Unable to give informed consent

Study Setting

Participants were recruited from seven sites, to include patients treated at both local units and cancer referral centres.

Sampling

Due to the sensitive nature of bladder cancer, in-depth, semi-structured qualitative interviews were undertaken. We aimed for maximum variation in our sampling, with participants selected on the basis of socio-demographic factors (age, gender, experience of the intervention(s), geographic spread, and time since treatment). A sample of approximately 24 to 30 patients was expected.

Sample identification and consent process

Patients fitting the inclusion criteria were identified by the clinical team from clinic databases and an approach made in person, by telephone, or by post. Patients were also identified at regular clinic appointments and an information pack provided and verbal consent sought for the patient's details to be passed to the research team. At least 48 hours was given between being given the information pack and the phone call from the research team. If no response was received, a reminder letter was sent 14 days after the date of the first letter. If no response was received to the second request, no further contact was made.

When an approach was made by post, a pack containing a letter, demographics form, PIS, expression of interest form, consent form and freepost envelope was sent to the patient inviting them to participate. On return of the expression of interest (EoI) slip and demographics form, patients were contacted by the research team to discuss the study. Once consent has been received, patients were contacted to set up an appointment. For telephone interviews, a copy of the consent form was signed by the researcher and posted to the participant. For face-to-face interviews, a copy of the signed consent form was given back to the participant on the day of the interview.

Patients were offered more time to consider participation and a number was provided that patients could use to contact the researcher. This recruitment strategy was selected because it minimises response bias and potentially increases the methodological rigour of the research (11).

Interview procedure

In depth semi-structured interviews were conducted with participants to elicit their beliefs about the two treatment options, their route to diagnosis, and to understand treatment burden and quality of life following treatment. A key role of the study was to understand and try to address issues around clinical trial participation, so we asked about likely trial burden, barriers to participation, recommendations for optimal recruitment and views about randomisation. Interviews were expected to last 45- 60 minutes. A topic guide was developed from the existing literature and discussions with the Chief Investigator, clinicians and Patient and Public Involvement members. Interviews were conducted by an experienced qualitative researcher. Since several studies (12, 13) show that there are no major differences in the results of telephone and face-to-face interviews, participants were given the option of a telephone interview to accommodate family and professional obligations. Interviews will be audio-recorded, with the permission of the participant.

Data analysis (as Phase 1 above)

Interviews were professionally transcribed verbatim and managed using NVivo. Personally identifiable data was removed or de-identified during transcription, and pseudonyms used. The data was analysed using Framework analysis (14) by three researchers independently coding the first three transcripts using initially inductive then deductive approaches. Codes and themes were compared after the analysis of the first three transcripts. Two researchers (AE & JB) then coded the remaining transcripts, with regular meetings with MT to ensure coding remains consistent. The analysis was further refined by using a constant comparison and contrastive approach, and looking for negative cases in order to examine for similarities and differences within and between patient groups.

Phase 3: Development of Training Package

The training package was developed from the findings of the interview and focus group data, and informed by the existing literature (9, 10). Training was delivered as a face-to-face

workshop delivered at 3 sites and incorporated presentations and role play exercises with simulated patients (trained individuals who are regularly used in communication skills training throughout healthcare education) (15, 16). A manual was developed to accompany the training and included: detailed information about the trial and the two treatments, information on how to discuss uncertainty (of treatment options), how to describe randomisation, how to talk to patients who express a treatment preference. The aim of the training day was to allow staff to practice their communication skills in relation to the trial and receive feedback.

Results

The findings of the work are currently being written up for publication.

Ethical issues

Confidentiality

We were mindful of protecting participant confidentiality at all times. Audio recordings were stored on a secure drive and accessed only by the researcher team. After analysis the audio recordings were destroyed. Personally identifiable data was removed during transcription and pseudonyms adopted; these bear no resemblance to the patient's identity, hospital number, DOB or similar. Participants were asked to consent to direct quotes. Paper documents (e.g. consent forms, demographic questionnaires etc.) are kept in a secure office, and electronic information stored on University computers which are password protected. The file in which codes are linked to patients' names is stored on a password protected computer on a secure network. All data will be archived in accordance with University of Leeds and University of Sheffield NHS Foundation Trust procedures.

Informed consent

The patients were required to sign a consent form prior to getting involved to the sub-study. Those unable to consent for themselves were excluded from participating.

Time frame: October 2015 to September 2016.

Patient and Public Involvement

One lay member (PK), was involved in the development of the proposal. PK was involved in the design of the study, and has commented on the wording of this protocol, as well as the PIS, consent forms and topic guides used in this study. PK will remain involved in the study. A patient group was set up for the project and provided input into the study at key points in the project (study design, development of training manual, data analysis, and dissemination). Lay members participated in the training events to co-deliver the training package.

REC Review and reports

Approval for the study was sought and obtained (REF 15/LO/1864) and the study obtained R & D approvals from the NHS Trusts involved.

External Peer Review

This study is funded by Yorkshire Cancer Research and has undergone independent expert peer review, including review by a qualitative methodologist and a clinician.

References

1. Lane JA, Donovan JL, Davis M, Walsh E, Dedman D, Down L, et al. Active monitoring, radical prostatectomy, or radiotherapy for localised prostate cancer: study design and diagnostic and baseline results of the ProtecT randomised phase 3 trial. The Lancet Oncology. 2014;15(10):1109-18.
2. Moynihan C, Lewis R, Hall E, Jones E, Birtle A, Huddart R, et al. The Patient Deficit Model Overturned: a qualitative study of patients' perceptions of invitation to participate in a randomized controlled trial comparing selective bladder preservation against surgery in muscle invasive bladder cancer (SPARE, CRUK/07/011). Trials [Electronic Resource]. 2012;13:228.
3. Kaur G, Hutchinson I, Mehanna H, Williamson P, Shaw R, Tudur Smith C. Barriers to recruitment for surgical trials in head and neck oncology: a survey of trial investigators. BMJ Open [Internet]. 2013.
4. Biedrzycki BA, editor Decision making for cancer clinical trial participation: a systematic review. Oncology nursing forum; 2010.
5. NIHR-CRNCC. New from the Network [webpage]. 2011 [cited 2015]. Available from: http://www.crncc.nihr.ac.uk/Resources/NIHR%20CRN%20CC/Documents/NFTNW%20Issue%202%20Qtr%202%202010.pdf.
6. Mills EJ, Seely D, Rachlis B, Griffith L, Wu P, Wilson K, et al. Barriers to participation in clinical trials of cancer: a meta-analysis and systematic review of patient-reported factors. The Lancet Oncology. 2006;7(2):141-8.
7. Hussain-Gambles M, Leese B, Atkin K, Brown J, Mason S, Tovey P. Involving South Asian patients in clinical trials. 2004.
8. Fletcher B, Gheorghe A, Moore D, Wilson S, Damery S. Improving the recruitment activity of clinicians in randomised controlled trials: a systematic review. BMJ open. 2012;2(1):e000496.
9. Mills N, Blazeby J, Hamdy F, Neal DE, Campbell B, Wilson C, et al. Training recruiters to randomised trials to facilitate recruitment and informed consent by exploring patients' treatment preferences. Trials [Electronic Resource]. 2014;15(323).
10. Realpe A, Adams A, Wall P, Griffin D, Donovan JL. A new simple six-step model to promote recruitment to RCTs was developed and successfully implemented. Journal of clinical epidemiology. 2016;76:166-74.
11. Hewison J, Haines A. Confidentiality and consent in medical research: overcoming barriers to recruitment in health research. BMJ: British Medical Journal. 2006;333(7562):300.
12. Novick G. Is there a bias against telephone interviews in qualitative research? Research in nursing & health. 2008;31(4):391-8.
13. Sturges JE, Hanrahan KJ. Comparing telephone and face-to-face qualitative interviewing: a research note. Qualitative Research. 2004;4(1):107-18.
14. Ritchie J, Spencer L, O'Connor W. Carrying out qualitative analysis In: Ritchie J, Lewis J, editors. Qualitative Research Practice: A guide for social science students and researchers. London: Sage; 2003. p. 219-62.
15. Wykurz G, Kelly D. Developing the role of patients as teachers: a literature review. BMJ. 2002;325(818-21).
16. Bokken L, Rethans J, Scherpbier A, Van der Vieuten C. Strengths and weaknesses of simulated and real patients in the teaching of skills to medical students: a review. Simulation in Healthcare: The Journal of the Society of Medical Simulation. 2008;3:161-69.
