# Supplementary material for: Radical cystectomy (bladder removal) against intravesical BCG immunotherapy for high-risk non-muscle invasive bladder cancer (BRAVO): a protocol for a randomised controlled feasibility study
Source: BMJ Open. 2017 Aug 11;7(8):e017913. doi: 10.1136/bmjopen-2017-017913 (PMC5724134; doi:10.1136/bmjopen-2017-017913)
Supplement: Author's manuscript [file bmjopen-2017-017913.draft_revisions.pdf]

Radical Cystectomy (Bladder Removal) against intra-vesical BCG immunotherapy for high risk non-muscle invasive bladder cancer (BRAVO): Protocol for a randomised controlled feasibility study

Journal:	*BMJ Open*
Manuscript ID	bmjopen-2017-017913
Article Type:	Protocol
Date Submitted by the Author:	24-May-2017

Complete List of Authors:	Oughton, Jamie; University of Leeds, Leeds Institute of Clinical Trials Research<br>Poad, Heather; University of Leeds, Leeds Institute of Clinical Trials Research<br>Twiddy, Maureen; University of Leeds, Leeds Institute of Health Sciences<br>Collinson, Michelle; University of Leeds, Leeds Institute of Clinical Trials Research<br>Hiley, Victoria; University of Leeds, Leeds Institute of Clinical Trials Research<br>Gordon, Kathryn; University of Leeds, Leeds Institute of Clinical Trials Research<br>Johnson, Mark; Freeman Hospital<br>Jain, Sunjay; St James's University Hospital<br>Noon, Aidan; University of Sheffield Medical School, Academic Urology Unit<br>Chahal, Rohit; Bradford Teaching Hospitals NHS Foundation Trust<br>Simms, Matt; Hull and East Yorkshire Hospitals NHS Trust<br>Dooldeniya, Mohantha; Mid Yorkshire Hospitals NHS Trust<br>Koenig, Phillip; Airedale NHS Foundation Trust<br>Goodwin, Louise; University of Sheffield Medical School, Academic Urology Unit<br>Brown, Julia; University of Leeds, Leeds Institute of Clinical Trials Research<br>Catto, James; University of Sheffield Medical School, Academic Urology Unit

**Primary Subject Heading**:	Oncology
Secondary Subject Heading:	Surgery, Urology
Keywords:	High risk non muscle invasive bladder cancer, HRNMIBC, mBCG, radical cystectomy, feasibility study, bladder cancer

SCHOLARONE™ Manuscripts

Radical Cystectomy (Bladder Removal) against intra-vesical BCG immunotherapy for high risk non-muscle invasive bladder cancer (BRAVO): Protocol for a randomised controlled feasibility study

Jamie B Oughton[1], Heather Poad[1], Maureen Twiddy[2], Michelle Collinson[1], Victoria Hiley[1], Kathryn Gordon[1], Mark Johnson[4], Sunjay Jain[5], Aidan P. Noon[3], Rohit Chahal[6], Matt Simms[7], Mohantha Dooldeniya[8], Phillip Koenig[9], Louise Goodwin[3], Julia M. Brown[1] and James W.F. Catto[3] for the BRAVO study group*

[1]Clinical Trials Research Unit, Leeds Institute of Clinical Trials Research, University of Leeds, Leeds, LS2 9JT, UK; [2]Leeds Institute of Health Sciences, University of Leeds, Leeds, UK; [3]Academic Urology Unit, University of Sheffield, Sheffield, UK; [4]Freeman Hospital, Newcastle, UK; [5]St James's University Hospital, Leeds, UK; [6]Bradford Teaching Hospitals NHS Foundation Trust, Bradford, UK; [7]Hull and East Yorkshire NHS Trust, Hull, UK; [8]Mid Yorkshire Hospitals NHS Trust, Wakefield, UK; [9]Airedale NHS Foundation Trust, Keighley, UK.

Corresponding Author:

Correspondence to;

James Catto, Academic Urology Unit, G Floor, The Medical School,

University of Sheffield, Beech Hill Road, Sheffield, S10 2RX, United Kingdom

Tel: +44 (0)114 226 1229

Fax: +44 (0)114 271 2268

Email: j.catto@sheffield.ac.uk

Word count: 5370

* BRAVO study group members: Zahir Abbasi, Linzi Bone, Angela Brocklehurst, Nicolas Bryan, Lindsay Caygill, Russsell Dowde, Jan Farrell, Jonathan Gill, Lisa Gledhill, Susan Hill, Helen Hothersall, Anne Kay, Phil Kelly, Bernadette Kilbane, Sanjeev Kotwal, Kate

For peer review only

For peer review only - http://bmjopen.bmj.com/site/about/guidelines.xhtml

Linton, Stephen Littler, Tiago Mendonca, Suzanne Miles, Stephen Mitchell, Peter Murphy, Vicky Murray, Naledi Mzwimbi, Alan Paul, Ramanan Rajasundaram, Julie Rawlings, Hannah Roberts, Helen Robertshaw, Wendy Robson, Derek Rosario, Kully Sandhu, Paul Sagar, Alison Shaw, Rajindrah Singh, Nick Smith, Tina Soar, Zoe Storton, Beverley Taylor, Francis Thomas, Nicky Thomas, Suresh Venugopal, Rachel Walker, Louise Weatherley, and Susan Wormley. Independent steering group members: Alison Birtle (Chair), Simon Fulford and Chris Metcalfe.

For peer review only

For peer review only - http://bmjopen.bmj.com/site/about/guidelines.xhtml

ABSTRACT

Introduction: High risk non-muscle invasive bladder cancer (HRNMIBC) is a heterogeneous disease which can be difficult to predict. Whilst around 25% of cancers progress to invasion and metastases, the remaining majority of tumours remain within the bladder. It is uncertain whether patients with HRNMIBC are better treated with intravesical maintenance BCG (mBCG) immunotherapy or primary radical cystectomy (RC). A definitive randomised controlled trial (RCT) is needed to compare these two different treatments, but may be difficult to recruit to and has not been attempted to date. Before undertaking such an RCT it is important to understand whether such a comparison is possible and how best to achieve it.

Methods and analysis: BRAVO is a multi-centre, parallel-group, mixed-methods, individually randomised, controlled, feasibility study for patients with HRNMIBC. Participants will be randomised to receive either mBCG immunotherapy or RC. The primary objective is to assess the feasibility and acceptability of performing the definitive phase III trial via estimation of eligibility and recruitment rates, assessing uptake of allocated treatment and compliance with mBCG, determining quality of life questionnaire completion rates and exploring reasons expressed by patients for declining recruitment into the study. We aim to recruit 60 participants from 6 centres in the UK. Surgical trials with disparate treatment options find recruitment challenging from both the patient and clinician perspective. By building on the experiences of other similar trials through implementing a comprehensive training package aimed at clinicians to address these challenges (qualitative sub study), we hope that we can demonstrate that a phase III trial is feasible.

For peer review only - http://bmjopen.bmj.com/site/about/guidelines.xhtml

Ethics and dissemination: The study has ethical approval (16/YH/0268). Findings will be made available to patients, clinicians, the funders, and the NHS through traditional publishing and social media.

Trial Registration: ISRCTN12509361 Registered 06/09/16.

Keywords: High risk non-muscle invasive bladder cancer, BCG, radical cystectomy, feasibility study, bladder cancer, surgical trial, RCT

For peer review only

For peer review only - http://bmjopen.bmj.com/site/about/guidelines.xhtml

Strengths and limitations of this study

- The study will determine the feasibility of randomising patients with HRNMIC, at multiple centres, to either radical cystectomy or intravesical maintenance BCG.
- This is an important comparison that has not been attempted before.
- This study will not determine which intervention is the superior treatment.
- A definitive phase III trial will need to be conducted to answer this question.
- Recruitment may be challenging and may not be possible through traditional care pathways.

For peer review only - http://bmjopen.bmj.com/site/about/guidelines.xhtml

INTRODUCTION

Context

Bladder cancer (BC) is a common disease that is one of the most expensive malignancies to manage[1]. Around 25% of patients present with poorly differentiated, low stage tumours; termed 'high risk non-muscle invasive bladder cancer' (HRNMIBC; including tumours with carcinoma in situ, invasion into the lamina propria and intra-epithelial spread into the prostatic urethra). The two main treatment options for HRNMIBC are intra-vesical immunotherapy (using a maintenance regime of intravesical *bacillus Calmette-Guerin* (mBCG)) and radical cystectomy (RC). The former aims to induce an immune response against the tumour and may reduce the risk of progression to muscle invasion[2]. Whilst mBCG avoids bladder removal, it leaves patients at risk of local progression and may impact upon quality of life (QoL) through local symptoms and anxiety. RC removes the risk of local disease progression and may have the best oncological outcomes, but could be overtreatment for non-progressing tumours. Many patients develop short-term post-operative complications after RC and others have a reduction in QoL following surgery. To date, RC and mBCG have not been directly compared. Their comparative risks and benefits are unknown, hampering decision-making, clinical care and exposing patients to both over and under-treatment.

Current knowledge

The natural history of HRNMIBC is unpredictable. Rates of progression to muscle invasion and metastases vary between 25-75%[3] and long term outcomes suggest around 20-25% of patients with HRNMIBC may die from BC[4 5]. mBCG avoids bladder removal and meta-analyses report potential reductions in progression by 5% at 2.5 years[6]. However, mBCG can be poorly tolerated, its impact upon progression is debated[2] and there are manufacturing

For peer review only

problems[7]. mBCG involves 27 intravesical instillations and 10 cystoscopies over 3 years. Many (74%) patients report local and systemic toxicity[8 9], so only 30% of patients complete mBCG[9 10]. Furthermore, there are few data to support that mBCG with bladder preservation preserves a good quality of life (QoL). With regards to oncological outcomes, reports of BC's-failing mBCG find upstaging to invasion in 27-63% of tumours and the cancer specific survival is worse than for BC with *de novo* muscle invasion (e.g. 37% vs 67%/3 years)[11-15]. RC includes removal of the bladder and adjacent organs, and reconstruction of urinary drainage. Many patients develop short-term bowel, respiratory or cardiovascular problems, including up to 20% require intervention[16]. Prospective studies report recovery of QoL following RC takes 6 months or longer to recover to pre-operative levels[17]. Recurrence-free survival rates following primary RC for HRNMIBC cancers appear superior to those from mBCG (e.g. 79%/10-years)[18].

Surgical RCTs

As contemporary data challenge the role of mBCG[2] and lessons have been learnt from large surgical RCTs[19], we believe it is time to compare mBCG with RC. This is an important comparison and this opportunity may be lost as RC for HRNMIBC becomes more popular[20]. Importantly, the 2015 NICE Bladder cancer guidelines selected this comparison as one of the highest ranked research priorities in the disease[21]. The BRAVO study aims to compare surgical and non-surgical treatments. Trials of similarly disparate treatments in BC have previously failed to recruit (e.g. CRUK-SPARE trial)[22 23]. Here we propose the preliminary work necessary to understand if we can undertake a large RCT of mBCG versus RC. Anticipated barriers to recruitment include patient and clinician-preferences, BC treatment pathways, a lack of high quality information[24 25], and the need for staff training in equipoise

For peer review only

For peer review only - http://bmjopen.bmj.com/site/about/guidelines.xhtml

and communicating RCT methods[26]. To address these issues, we will develop a tailored staff training package to facilitate informed decision making about participation and to better understand RCT methodology. The development work will be informed by existing knowledge[24 27 28] and context-specific evidence derived from interviews with patients and healthcare staff exploring: a) treatment perceptions, b) patient pathways to treatment; c) barriers to participation, d) training needs of site staff. This qualitative work to develop and deliver the training package is described in a separate protocol (Supplementary File 1). We will then undertake a feasibility study to assess whether recruitment could be achieved in a definitive trial, embedding a qualitative component to establish patient experience.

Study Aims

Our aims are to assess whether a larger phase III RCT is possible and to acquire sufficient data to aid planning such a trial. Primary outcomes are:

1. To assess the number of patients screened and identified as eligible within these 6 centres.
2. To assess recruitment rates (number of patients randomised per month).

Secondary outcomes are:

1. To assess acceptance of allocated treatment.
2. To assess the rate of compliance with mBCG at 12 months after randomisation and collect reasons for non-compliance.
3. To assess the feasibility and optimal frequency of collecting QoL data in patients treated for HRNMIBC.
4. To obtain preliminary data on the QoL data of patients treated for HRNMIBC.
5. To explore the reasons expressed by patients for declining recruitment into the study.

For peer review only

METHODS AND DESIGN

Trial Design

BRAVO is a multi-centre, parallel-group, mixed-methods, individually randomised, controlled feasibility study in patients with HRNMIBC suitable for treatment by either mBCG or RC. Eligible, consenting patients will be randomised (1:1) to receive either mBCG or RC (Figure 1). Due to the different treatment modalities in the two arms, it is not feasible to blind patients or clinicians to treatment allocation. Patient reported outcome data will be collected at 3, 6 and 12-months post-randomisation in clinic or by postal questionnaire if the patient is not due to attend a clinic visit.

Trial Population

We aim to recruit 60 patients from 6 UK cancer centres and their associated District General Hospitals. The inclusion criteria are:

1. Male or female aged ≥ 18 years old.
2. Patients with a new diagnosis of high-risk (high grade[29] or grade 3[30]) non-muscle invasive urothelial carcinoma (staged as either pTa, pTis or pT1). Patients with previous low grade non muscle invasive bladder cancer (NMIBC) are eligible.
3. The tumour is either solely urothelial cell carcinoma or has urothelial cell carcinoma as the majority histological component.
4. In addition to the HRNMIBC bladder tumour, there needs to be one or more risk factor from:
    a. Presence of pTis in the bladder
    b. Presence of pTis in the prostatic urethra
    c. Lymphovascular invasion

For peer review only

For peer review only - http://bmjopen.bmj.com/site/about/guidelines.xhtml

d. Vascular invasion

e. Residual Grade 3/High grade UCC on re-resection (or initial TURBT if no re-resection)

f. Multifocal disease (>3 tumours at initial resection)

g. Young age (<65 years old)

h. Initial tumour Size > 3cm (or >5g in histology specimen)

i. pT1 stage

5. *Either* re-resection of the bladder (following the initial diagnostic TURBT) within the 3 months prior to randomisation confirming the absence of muscle invasion

OR

a. the initial diagnostic TURBT biopsy contains muscle, AND

b. the radiological and pathological stage assessment are in agreement regarding stage and absence of muscle invasion, AND

c. a re-resection is not appropriate in the opinion of the treating clinician AND

d. the initial TURBT is within 3 months prior to randomisation.

6. CT or cross sectional imaging of the abdomen and pelvis within the year prior to starting treatment.

7. Imaging of the lungs and thorax within 3 months prior to randomisation.

8. Suitable and fit for both mBCG and RC as determined by the treating clinician.

9. Central MDT pathological review agrees diagnosis.

10. If female, must be (as documented in patient notes):

a. postmenopausal (no menses for 12 months without an alternative medical cause), or

For peer review only

For peer review only - http://bmjopen.bmj.com/site/about/guidelines.xhtml

b. surgically sterile (hysterectomy, bilateral salpingectomy or bilateral oophorectomy), or
c. using acceptable contraception (which must be continued for 7 days after the last dose of BCG or until RC is carried out). Women of child bearing potential must undergo a pregnancy test before randomisation.
d. not breast feeding.

The exclusion criteria are:

1. Solely non-urothelial or any variant urothelial pathology
2. Unable or not willing to give informed consent
3. Previous high risk (high grade or grade 3) NMI or invasive bladder cancer
4. Any previous treatment with intravesical BCG
5. Any previous treatment with pelvic radiotherapy
6. Any other malignancy (excluding non-melanomatous skin cancer, low-risk prostate cancer and prior low risk bladder cancer)

Eligibility waivers are not permitted.

Prior to entry, patients must be accurately staged (e.g. cross sectional imaging (e.g. CT) of the abdomen, pelvis and thorax, or bone scan if indicated, within 3 months prior to randomisation) and judged to be eligible for both treatments (anesthetic evaluation in those with borderline fitness for RC). After trial entry, women of childbearing age must be proven to be not pregnant (pregnancy test).

Sample Size

For peer review only

For peer review only - http://bmjopen.bmj.com/site/about/guidelines.xhtml

The sample size for this feasibility study has been set to give confidence that the recruitment target for the main trial can be met. A formal power calculation is not appropriate as effectiveness is not being evaluated. It is estimated that per year, over the six centres there will be approximately 1000 new diagnoses of NMIBC, where 20% are likely to be eligible (200 patients)[31]. We would need to show that we are able to randomise approximately 25% of all eligible patients to be confident that the recruitment target for the main trial would be met within 3 years, with an additional 9 centres. We therefore plan to recruit 60 patients over an 18 month period in the feasibility study. For the phase III trial, we anticipate either a single primary endpoint (cancer-specific survival) or co-primary endpoints (cancer-specific survival and averaged QALYs). We estimate 506 participants are required to have 80% power to show a superiority hazard ratio of 0.626 (based on an improvement in 5-year cancer specific survival from 70% in the BCG arm to 80% in the RC arm), assuming a 3-year accrual period, 5 years of follow-up, and accounting for 5% loss-to-follow-up.

Setting

Participants will be recruited from 6 cancer centres (and 7 neighbouring district hospitals) within Yorkshire and Northumberland. NHS demographic data show that Yorkshire and Northumberland have some of the highest rates of BC incidence and some of the lowest rates of survival from this cancer[32 33].

Recruitment

Patients will be identified through multidisciplinary team (MDT) meetings and approached once they know their diagnosis of HRNMIBC. This approach may be at any hospital involved in their care and by medical or nursing staff. The team will introduce the trial when treatment options are being discussed, provide the introduction leaflet and ask permission

For peer review only - http://bmjopen.bmj.com/site/about/guidelines.xhtml

(and contact details) for a Research Nurse to contact the patient with more information. The number of eligible and screened patients will be recorded. Interested participants will be invited to attend an appointment at the research site and/or receive telephone calls, to be given a full explanation of the BRAVO study. Experience in similar studies suggests patients can be overwhelmed by information given in clinic, and that telephone contact can help and provides another opportunity to support patients. Up to five attempts will be made to contact the participant by telephone, after which it will be assumed they have decided to not participate. Eligible patients can be contacted by post if the immediate care team deem this best. No contact information will be shared outside of the team directly caring for the patient unless consent has been obtained.

Consent

Informed consent takes place in a face to face setting at the research site. Patients will have at least 24 hours to consider participation and will be encouraged to discuss the study with their family and other healthcare professionals. A full verbal explanation of the study, a written PIS (Patient Information Sheet detailing rationale, design and personal implications of trial entry) and informed consent form (CF) will be provided. Participants may withdraw at any stage of the trial. Consent will be obtained prior to collection of baseline assessment data and subsequent randomisation.

Staff training

We recognise the challenge of comparing these two treatment choices and that the patient pathway includes interaction with numerous healthcare providers. To minimise bias and to maintain equipoise, a training package will be developed from interviews with patients and clinicians and delivered to staff who are likely to care for patients before and during the

For peer review only

study. Training will incorporate lectures and role play exercises with simulated patients. A careful explanation of the potential risks and benefits of the two treatment interventions is crucial, such risks will be clearly explained to interested patients in an unbiased and fair way, assisted by written study-specific patient information.

Randomisation

Patients will be randomised, using a 24-hour centralised telephone or web based randomisation system, on a 1:1 basis to receive either RC or mBCG. A computer-generated adaptive minimisation algorithm that incorporates a random element will be used to ensure the treatment groups are balanced (stratified) for:

- Age (<75, >=75)
- Sex (male, female)
- Recruiting cancer centre
- Tumour stage (pTa/pTis, pT1)
- Presence of carcinoma in situ (Yes, No)
- Previous low risk bladder cancer (Yes, No)

Intervention - BCG immunotherapy

Maintenance BCG immunotherapy will be administered at either the cancer centre or district general hospital using the SWOG protocol[10]. At least 12 months of BCG treatment are required and 6 weeks of induction BCG will be followed by 3 doses at 4 and 10 months after diagnosis. Delays and deferrals are common and allowed within this study. BCG induction should include at least 4 (of 6) doses of BCG and induction should be completed within 10 weeks. The presence of an invasive BC requires the cessation of mBCG and a change in

For peer review only

For peer review only - http://bmjopen.bmj.com/site/about/guidelines.xhtml

treatment intent. Maintenance BCG may continue in the presence of low risk NMI and HRNMI bladder cancer at the first cystoscopy, thereafter these are managed as recurrences and require patient discussion. Rigid cystoscopy with bladder biopsy and bladder washings is mandated at the first check. After this, bladder surveillance is performed as per local protocol (flexible or rigid instruments). All cystoscopies will be undertaken or directly supervised (with a visual check) by a Consultant Urologist who manages HRNMIBC. Fluorescence or narrow band imaging may be used, as per local protocols. Histological review of the bladder biopsies and urinary cells should be performed to determine the presence or absence of BC.

Local and systemic complications are common in mBCG regimens and should be managed as per local protocol. The study will collect data on the frequency of expected BCG toxicities and whether this leads to the cessation of BCG treatment. Cystectomy may be performed within BRAVO for severe BCG-related toxicities, if these warrant such an intervention. Patients undergoing BCG treatment may stop treatment due to disease progression, disease recurrence, serious BCG intolerance or side effects or patient choice. Disease progression: patients who have confirmed progressive disease after any of the check cystoscopies (presence of pT2 tumours, cancer in lymph nodes, or metastases) should stop BCG and be offered curative treatment for muscle invasive bladder cancer. Disease recurrence is defined as the presence of low risk NMI or HRNMIBC from the second check cystoscopy onwards. Participants with recurrence should be offered the option of changing treatment, including radical cystectomy or using second line intravesical approaches.

Intervention - Radical Cystectomy

Radical cystectomy should be performed at each cancer centre by teams specialising in this service. Variations in surgical performance and practice produce wide differences in

For peer review only

For peer review only - http://bmjopen.bmj.com/site/about/guidelines.xhtml

morbidity and mortality from RC[34]. To mitigate these, surgeons within BRAVO will have individually undertaken at least 10 RCs per year for the last 2 years (or 20 in the last year), have median length of stay rates under 16 days, have 90-day post-RC mortality rate of less than 10% (collected outcomes from the British Association of Urological Surgeons (BAUS) RC complex dataset[31]). Post-operative complication rates and intra and post-operative transfusion rates will also be taken into consideration. Individual surgeon data will act as surrogate measures for the entire surgical team and require accreditation from the Trial Management Group before entry into BRAVO. Submitted data for surgical accreditation should reflect the practice to be undertaken within this study (e.g. open or robotic approaches). Surgery should take place within 8 weeks of randomisation.

Cystectomy should include removal of adjacent organs. In males, this includes the prostate and seminal vesicles. In females, this should include a section of adjacent anterior vaginal wall, the uterus, cervix and fallopian tubes and, if no bladder reconstruction is planned, the urethra. Oophorectomy is optional, as per local practice and individualised for each patient. Pelvic lymphadenectomy is mandated within BRAVO. The template should at least include the regional lymph nodes up to the level of the ureteric crossing of the common iliac vessels. This includes the obturator fossa, the external iliac and internal iliac nodes. A more extended lymphadenectomy is acceptable. Excised lymphatic tissue should be submitted for histological analysis. Perioperative care is to be carried out as per Enhanced Recovery After Surgery (ERAS) protocols[35 36].

Withdrawal of treatment

In line with usual clinical care, cessation or alteration of regimens will be at the discretion of attending clinicians or the participants. All participants who withdraw or are withdrawn from

For peer review only - http://bmjopen.bmj.com/site/about/guidelines.xhtml

their allocated treatment will still attend for follow-up assessments and complete questionnaires unless unwilling to do so and outcomes will continue to be collected. In the event that a patient withdraws consent prior to randomisation, data collected up to the point of withdrawal will be analysed.

Data collection

A screening form, to include demographic details and reasons for ineligibility, exclusion or refusal, will be completed for all patients considered for BRAVO. A feedback questionnaire will be used to identify patients who are willing to take part in the qualitative sub study (Supplementary File 1). Baseline assessments prior to randomisation include QoL scores (EuroQuol-5D (EQ-5D) [37], EORTC QLQ-C30 [38], EORTC QLQ-BLM30) at trial entry.

Within mBCG, outcomes and compliance data will be collected at each cystoscopy. For RC, patient and operative data will be collected at the time of surgery, as per our national register [31], and then at each subsequent follow up visit (3, 6 and 12 months post randomisation). Follow up imaging (CT scan) to assess response to treatment will be performed in both arms at one year post randomisation. QoL questionnaires will be collected at 3, 6 and 12 months post-randomisation in face to face consultations or by telephone. These include EuroQuol-5D (EQ-5D) [37], EORTC QLQ-C30 [38], and either EORTC QLQ-BLM30 (for those randomised to RC) or EORTC QLQ-NMIBC24 (for those randomised to BCG). Information will be collected on deaths, complications and toxicities (adverse events), and related and unexpected serious adverse events up to one year post randomisation, or three months after the last participant is randomised if earlier.

For peer review only

Statistical analyses

A detailed statistical analysis plan will be written before any analysis is undertaken. All analyses and data summaries will be conducted on the intention-to-treat (ITT) population. No formal interim analyses are planned and final analysis will take place when all available data have been received. The analysis will focus on descriptive statistics and confidence interval estimation. Primary analysis will include summaries of the number of patients at each stage of the recruitment pathway (screening, eligibility, consent and randomisation) and assessment of the overall monthly recruitment rate. Secondary analysis will include summaries of acceptance of randomised treatment and mBCG treatment compliance. Participant retention and self-reported QoL outcomes during follow-up, including withdrawal data (timing and reason), will also be summarised overall and by time-point. Levels of missing data in QoL outcomes will be assessed. The median cancer-specific survival estimate and its corresponding 95% confidence interval (CI) will be calculated to inform the sample size calculation of the phase III trial. As this is to aid the design of a pragmatic phase III trial, all randomised patients will be included in the calculation, regardless of treatment received. Cancer-specific survival will be calculated from the date of randomisation to the date of cancer-specific death. Participants with missing follow-up data, or who are alive at the time of the analysis will be censored at the date they were last known to be alive. Overall survival, calculated from the date of randomisation to the date of death, will also be summarised as for cancer-specific survival.

The frequent collection of QoL data within this feasibility study is necessary in order to assess the burden to patients. This will be assessed by monitoring collection compliance rates and will inform the optimal frequency of data collection for the main trial. Averaged QALYs

For peer review only - http://bmjopen.bmj.com/site/about/guidelines.xhtml

may be a co-primary endpoint for the main trial, as such, determining the optimal frequency of EQ-5D data collection within this feasibility study is crucial.

Safety

The number of AEs and RUSAEs will be summarised descriptively by arm, by grade, and body system. The proportion of participants experiencing each toxicity will be summarised by maximum NCI CTCAE grade[39] experienced, overall and by arm. Operative RC complications will be graded using the Clavien Dindo classification[40].

Criteria for progression to the definitive phase III trial

The following guidelines for progression to a definitive phase III trial have been defined:

- The recruitment and follow-up rates must demonstrate that a definitive trial using similar procedures will achieve sufficient power to test the hypothesised difference between treatment arms.
- The sample size calculation for the feasibility study and proposed phase III trial are provided earlier. This assumes that 20% of all new diagnoses of NMIBC would be eligible and approximately 25% of those would be randomised. To proceed to a definitive trial, we need to show that at least 20% of eligible patients can be randomised.

Qualitative sub study

There are two qualitative studies. The first was undertaken prior to the start of the RCT to identify a priori the barriers to recruitment from the perspectives of patients and staff to inform the development of a bespoke training package for staff[41] (see Supplementary File 1). A second qualitative study is embedded into the RCT trial to understand patients' views and

For peer review only - http://bmjopen.bmj.com/site/about/guidelines.xhtml

experiences of the treatments and explore patients' acceptability of the study and recruitment processes:

Qualitative sub study objectives

1. To gauge patients understanding of the study and their views on the recruitment process.
2. To qualitatively explore patient's acceptability of the study to assist in optimisation of recruitment strategies employed for the definitive trial.
3. Explore reasons for participation and non-participation of eligible patients.
4. Understand patients' experience of the randomisation process on decision making.
5. Understand why people refuse to participate or do not take up allocated treatment.
6. Patient understanding of study materials i.e. do patients understand what will happen if they take part and do they understand what they are being randomised to.
7. Acceptability of study procedures.
8. Acceptability of randomisation.

Qualitative sub study overview

In order to examine the views and experiences of bladder cancer patients we will conduct in-depth semi-structured interviews with patients approached to take part in the trial. Qualitative findings will help illuminate the acceptability of trial processes and explore barriers to uptake.

Recruitment to RCTs with very different treatment arms can be difficult and recruitment to trials involving surgery is particularly challenging[42]. Trials present practical and methodological challenges, including difficulties in recruitment, randomisation and lack of clinical equipoise[43]. Understanding why patients do or do not participate in trials is important

For peer review only - http://bmjopen.bmj.com/site/about/guidelines.xhtml

and clinical trials have recently begun to incorporate a qualitative component to address these issues. These studies have been able to successfully identify aspects of the trial design that hindered recruitment and identify possible solutions[42 44].

Qualitative sub study design

All eligible patients will be asked to complete a questionnaire to gauge their understanding of BRAVO and their views on the recruitment process. We will collect data from patients who decline the study, who consent but refuse allocation and those who consent and accept allocation. A short questionnaire will be given to seek patient views on the recruitment process and to ask if participants would be willing to provide detailed feedback by face to face or telephone interview. A purposive sample of 15 patients will be selected for interview. Written consent will be taken prior to the interview and a flexible topic guide developed in conjunction with PPI representatives, clinical colleagues and informed by the literature used to assist questionning. The topic guide will be devised to ensure the key issues are covered but do not dictate data collection; and will be flexible enough to elicit participants own experiences and views of the trial as well as issues unanticipated by the interview team. Interviews will be audio-recorded, transcribed and anonymised to protect confidentiality. With their consent, participants may be contacted after the interview to answer questions which may emerge during the analysis, or to explore issues that emerged in the interviews in more depth.

Qualitative sub study data analysis

Qualitative data will be analysed by the qualitative researcher. Interview transcripts will be checked for accuracy and then managed using NVivo qualitative data analysis software (QSR International, Daresbury, UK) which aids the indexing of qualitative data. Analysis will start during data collection and will inform later data collection; for example emerging themes may

For peer review only - http://bmjopen.bmj.com/site/about/guidelines.xhtml

identify new questions to explore in later interviews. The data will be analysed using thematic analysis[45 46] using an inductive (bottom-up) approach to identify and analyse patterns across the data set using constant comparison methods[47 48]. Inductive coding will follow using a line-by-line coding approach, with codes assigned to segments of data which provide insight into participants' views of the trial. An initial coding frame will be developed from the first interviews and will be modified, if necessary, as the analysis develops. A subset of transcripts will be independently coded by another member of the team and compared to ensure consistency. Any discrepancies will be discussed with the research team and resolved to achieve coding consensus. The data will be examined for negative cases and the reasons explored by comparison with the overall dataset.

Data Monitoring

Trial supervision includes a core project team, a trial management group (TMG), and an independent Trial Steering Committee (TSC). For a feasibility study of this nature and duration, a separate data monitoring and ethics committee is not required; rather; the TSC adopts a safety monitoring role and will review safety issues if this becomes necessary. Data will be monitored for quality and completeness by the CTRU. Missing data (except individual items collected via questionnaires) will be chased until received, confirmed as not available or the trial is at analysis. Any protocol changes will be disseminated by the CTRU to the relevant parties.

Trial Organisation and Administration

The trial was developed by the BRAVO Trial Management Group (TMG). The trial is funded by Yorkshire Cancer Research and is sponsored by the Sheffield Teaching Hospitals NHS Trust (Clinical Research Office, Royal Hallamshire Hospital, D Floor, Glossop Road,

For peer review only - http://bmjopen.bmj.com/site/about/guidelines.xhtml

Sheffield), co-ordinated by the CTRU, University of Leeds, and is registered (ISRCTN12509361). The trial will be conducted in accordance with the principles of Good Clinical Practice in clinical trials, as applicable under UK regulations, the NHS Research Governance Framework and through adherence to CTRU standard operating procedures (SOPs). CTRU/sponsor have systems in place to ensure that serious breaches of GCP or the trial protocol are identified and reported. Ethical approval has been obtained from the National Research Ethics Service Committee Yorkshire & Humber – South Yorkshire (reference 16/YH/0268). Any on-site source data verification carried out by the CTRU is not independent from sponsor. Sheffield Teaching Hospitals NHS Trust will not be liable for negligent harm caused by the design of the trial. No additional compensation for clinical negligence will be provided for trial participants over that which is available to NHS patients. The results of the study will be published in peer-reviewed publications and will be presented at relevant national and international conferences. We will work with our patient panel of bladder cancer survivors to develop lay reports to disseminate research findings to patient groups and the clinical teams at participating sites.

ETHICS AND DISSEMINATION

The 2015 NICE bladder cancer guidelines identified the comparison between mBCG and RC as one of their highest research priorities[21]. This reflects the importance of this question, but does not address how randomisation between two very different treatment options should occur or whether such a comparison is possible. Within this feasibility study we are attempting to understand, address and develop methodology to allow such a comparison. This will require several key issues to be addressed. Firstly, it is clear from other surgical vs. non-surgical treatment trials[49] that the most important element for RCT recruitment is keeping

For peer review only

equipoise when discussing the treatment options by medical and nursing staff. Whilst previous studies used research nurses to keep equipoise, this is not viable across many centres within the current research funding climate. In an attempt to replicate this model we ran a number of educational days to train relevant medical and nursing staff about the importance of equipoise and to discuss their beliefs about HRNMIBC. All staff had opinions about the efficacy of BCG and the quality of life with RC, and so it was important to discuss these in an open forum to challenge these views and use evidence to dispel prior beliefs. We proposed a six-stage consultation plan to help staff keep patients at equipoise and so facilitate trial entry and treatment acceptance[50]. Within this feasibility study we will determine if this approach is possible and successful. Secondly, UK data do not accurately identify the number of patients with HRNMIBC, what proportion of these are suitable for both RC and mBCG, and how many of these would accept randomised treatment options. Within this feasibility study we will establish accurate data about the number of eligible cases across this population and understand what proportion accept their randomised treatment allocation. We will use these findings to power the phase III comparative study. Finally, there are very few reliable data about quality of life with mBCG and none that compare this directly to RC. Within this study we will produce these data within 60 patients (30 for each arm) and so allow this endpoint to be modelled for the larger phase III study.

Availability of data

The CTRU will control the final trial dataset and any requests for access will be reviewed by the TMG and TSC, subject to existing contractual arrangements with the funders. The protocol, sample case report forms and participant information are available on a case by case basis as agreed by the TMG, upon request to the corresponding author.

For peer review only - http://bmjopen.bmj.com/site/about/guidelines.xhtml

Trial Status

The trial opened to recruitment in October 2016 using protocol version 2.0 (08/08/2016) and is due to close in March 2018.

The protocol was amended to version 3 in October 2016 to account for additional inclusion and exclusion criteria, and updated surgeon accreditation criteria. The protocol was amended to version 4 in November 2016 to further update the inclusion criteria and surgeon accreditation criteria. Both amendments were reviewed and approved by the sponsor, and the National Research Ethics Service Committee Yorkshire & Humber – South Yorkshire (reference 16/YH/0268). Protocol amendments are disseminated to relevant parties by CTRU.

DECLARATIONS

Authors' Contributions

Conception and design of the BRAVO trial: JWFC, JBO, HP, VH, MC, JMB.

Protocol/Patient Information Sheet: JWFC, JBO, HP, MC, VH, MT, LG.

Writing of manuscript: JBO, JWFC, HP, MC, MT, KG, MJ, SJ, RC, MS, MD, PK, VH, LG, JMB.

All authors have read and approved the final manuscript. The trial will comply with the authorship criteria recommended by the International Committee of Medical Journal Editors.

For peer review only

For peer review only - http://bmjopen.bmj.com/site/about/guidelines.xhtml

Acknowledgements & funding

We gratefully acknowledge the ongoing support of participants, principal investigators, research nurses, MDT coordinators, data managers and other site staff who have been responsible for setting up, recruiting participants and collecting the data for the trial. This trial was funded by Yorkshire Cancer Research (Study S388) and we acknowledge the help of Kathryn Scott in the development of this project. The funder had no role in the design, analysis or collection of the data; in writing the manuscript; or in the decision to submit the manuscript for publication. We are grateful for the trial oversight provided by the sponsor Sheffield Teaching Hospitals NHS Trust and the members of the TSC.

Competing Interests

The other authors declare no other competing interests

For peer review only

For peer review only - http://bmjopen.bmj.com/site/about/guidelines.xhtml

REFERENCE LIST

1. Chavan S, Bray F, Lortet-Tieulent J, et al. International variations in bladder cancer incidence and mortality. European urology 2014;**66**(1):59-73.

2. Malmstrom PU, Sylvester RJ, Crawford DE, et al. An individual patient data meta-analysis of the long-term outcome of randomised studies comparing intravesical mitomycin C versus bacillus Calmette-Guerin for non-muscle-invasive bladder cancer. European urology 2009;**56**(2):247-56.

3. Sylvester RJ, van der Meijden AP, Oosterlinck W, et al. Predicting recurrence and progression in individual patients with stage Ta T1 bladder cancer using EORTC risk tables: a combined analysis of 2596 patients from seven EORTC trials. European urology 2006;**49**(3):466-5; discussion 75-7.

4. Kulkarni GS, Hakenberg OW, Gschwend JE, et al. An updated critical analysis of the treatment strategy for newly diagnosed high-grade T1 (previously T1G3) bladder cancer. European urology 2010;**57**(1):60-70.

5. Thomas F, Noon AP, Rubin N, et al. Comparative outcomes of primary, recurrent and progressive high-risk non-muscle invasive bladder cancer. European urology 2013;**63**(1):145-54.

6. Sylvester RJ, van der MA, Lamm DL. Intravesical bacillus Calmette-Guerin reduces the risk of progression in patients with superficial bladder cancer: a meta-analysis of the published results of randomized clinical trials. J Urol 2002;**168**(5):1964-70.

7. Mostafid AH, Palou Redorta J, Sylvester R, et al. Therapeutic options in high risk non-muscle invasive bladder cancer during the current worldwide shortage of Bacille Calmette Guerin. European urology 2015;**In Press**.

8. Lamm DL, van der Meijden PM, Morales A, et al. Incidence and treatment of complications of bacillus Calmette-Guerin intravesical therapy in superficial bladder cancer. J Urol 1992;**147**(3):596-600.

9. van der Meijden AP, Sylvester RJ, Oosterlinck W, et al. Maintenance Bacillus Calmette-Guerin for Ta T1 bladder tumors is not associated with increased toxicity: results from a European Organisation for Research and Treatment of Cancer Genito-Urinary Group Phase III Trial. European urology 2003;**44**(4):429-34.

10. Lamm DL, Blumenstein BA, Crissman JD, et al. Maintenance bacillus Calmette-Guerin immunotherapy for recurrent TA, T1 and carcinoma in situ transitional cell carcinoma of the bladder: a randomized Southwest Oncology Group Study. J Urol 2000;**163**(4):1124-9.

11. Lambert EH, Pierorazio PM, Olsson CA, et al. The increasing use of intravesical therapies for stage T1 bladder cancer coincides with decreasing survival after cystectomy. BJU Int 2007;**100**(1):33-6.

For peer review only - http://bmjopen.bmj.com/site/about/guidelines.xhtml

12. Schrier BP, Hollander MP, van Rhijn BW, et al. Prognosis of muscle-invasive bladder cancer: difference between primary and progressive tumours and implications for therapy. European urology 2004;**45**(3):292-6.

13. Huguet J, Crego M, Sabate S, et al. Cystectomy in patients with high risk superficial bladder tumors who fail intravesical BCG therapy: pre-cystectomy prostate involvement as a prognostic factor. European urology 2005;**48**(1):53-9; discussion 59.

14. Denzinger S, Fritsche HM, Otto W, et al. Early versus deferred cystectomy for initial high-risk pT1G3 urothelial carcinoma of the bladder: do risk factors define feasibility of bladder-sparing approach? European urology 2008;**53**(1):146-52.

15. Solsona E, Iborra I, Rubio J, et al. The optimum timing of radical cystectomy for patients with recurrent high-risk superficial bladder tumour. BJU Int 2004;**94**(9):1258-62.

16. Shabsigh A, Korets R, Vora KC, et al. Defining Early Morbidity of Radical Cystectomy for Patients with Bladder Cancer Using a Standardized Reporting Methodology. European urology 2009;**55**(1):164-74.

17. Hardt J, Filipas D, Hohenfellner R, et al. Quality of life in patients with bladder carcinoma after cystectomy: first results of a prospective study. Qual Life Res 2000;**9**(1):1-12.

18. Hautmann RE, Volkmer BG, Gust K. Quantification of the survival benefit of early versus deferred cystectomy in high-risk non-muscle invasive bladder cancer (T1 G3). World J Urol 2009;**27**(3):347-51.

19. Lane JA, Donovan JL, Davis M, et al. Active monitoring, radical prostatectomy, or radiotherapy for localised prostate cancer: study design and diagnostic and baseline results of the ProtecT randomised phase 3 trial. Lancet Oncol 2014;**15**(10):1109-18.

20. Stein JP, Penson DF. Invasive T1 bladder cancer: indications and rationale for radical cystectomy. BJU Int 2008;**102**(3):270-5.

21. NICE. Bladder cancer Guidelines. Secondary Bladder cancer Guidelines 2015. https://www.nice.org.uk/guidance/indevelopment/gid-cgwave0600/documents.

22. Huddart RA, Hall E, Lewis R, et al. Life and death of spare (selective bladder preservation against radical excision): reflections on why the spare trial closed. BJU Int 2010;**106**(6):753-5.

23. Paramasivan S, Huddart R, Hall E, et al. Key issues in recruitment to randomised controlled trials with very different interventions: a qualitative investigation of recruitment to the SPARE trial (CRUK/07/011). Trials 2011;**12**(78).

24. Moynihan C, Lewis R, Hall E, et al. The Patient Deficit Model Overturned: a qualitative study of patients' perceptions of invitation to participate in a randomised controlled trial comparing selective bladder preservation against surgery in muscle invasive bladder cancer (SPARE, CRUK/07/011). Trials 2012;**13**(228).

25. Kaur G, Smyth R, Williamson P. Developing a survey of barriers and facilitators to recruitment in randomised controlled trials. Trials 2012;**13**(218).

For peer review only

For peer review only - http://bmjopen.bmj.com/site/about/guidelines.xhtml

26. Fletcher B, Gheorghe A, Moore D, et al. Improving the recruitment activity of clinicians in randomised controleed trials. BMJ open 2012;**2: e000496**.

27. Mills N, Blazeby J, Hamdy F, et al. Training recruiters to randomized trials to facilitate recruitment and informed consnet by exploring patients' treatment preferences. Trials 2014;**15**(323).

28. Mills N, Donovan J, Wade J, et al. Exploring treatment preferences facilitated recruitment to randomised controlled trials. Journal of clinical epidemiology 2011;**64**:1127-36.

29. JN E, G S, JI E. *World Health Organization classification of tumours. Pathology and genetics of tumours of the urinary system and male genital organs*. Lyon, France: IARC Press, 2004.

30. *Histological typing of urinary bladder tumours*. Geneva, Switzerland: World Health Organization, 1973.

31. Boustead GB, Fowler S, Swamy R, et al. Stage, grade and pathological characteristics of bladder cancer in the UK: British Association of Urological Surgeons (BAUS) urological tumour registry. BJU Int 2014;**113**(6):924-30.

32. National Cancer Intelligence Network Cancer e-atlas. Secondary National Cancer Intelligence Network Cancer e-atlas. http://www.ncin.org.uk/cancer_information_tools/eatlas.

33. Thomas F, Rosario DJ, Rubin N, et al. The long-term outcome of treated high-risk non-muscle invasive bladder cancer: Time to change treatment paradigm? Cancer 2012;**118**(22):5525-34.

34. Konety BR, Dhawan V, Allareddy V, et al. Impact of hospital and surgeon volume on in-hospital mortality from radical cystectomy: data from the health care utilization project. J Urol 2005;**173**(5):1695-700.

35. Fearon KC, Ljungqvist O, Von Meyenfeldt M, et al. Enhanced recovery after surgery: a consensus review of clinical care for patients undergoing colonic resection. Clin Nutr 2005;**24**(3):466-77.

36. Azhar RA, Bochner B, Catto J, et al. Enhanced Recovery after Urological Surgery: A Contemporary Systematic Review of Outcomes, Key Elements, and Research Needs. European urology 2016;**70**(1):176-87.

37. Rabin R, de Charro F. EQ-5D: a measure of health status from the EuroQol Group. Ann Med 2001;**33**(5):337-43.

38. Aaronson NK, Ahmedzai S, Bergman B, et al. The European Organization for Research and Treatment of Cancer QLQ-C30: a quality-of-life instrument for use in international clinical trials in oncology. J Natl Cancer Inst 1993;**85**(5):365-76.

39. Common Terminology Criteria for Adverse Events (CTCAE) Version 4.0. http://evs.nci.nih.gov/ftp1/CTCAE/CTCAE_4.03_2010-06-14_QuickReference_5x7.pdf: U.S Department of Health and Human Services, National Institute of Health, 2009.

For peer review only - http://bmjopen.bmj.com/site/about/guidelines.xhtml

40. Dindo D, Demartines N, Clavien PA. Classification of surgical complications: a new proposal with evaluation in a cohort of 6336 patients and results of a survey. Ann Surg 2004;**240**(2):205-13.

41. Edmondson AJ, Birtwistle JC, Catto JW, et al. The patients' experience of a bladder cancer diagnosis: a systematic review of the qualitative evidence. J Cancer Surviv 2017.

42. McDonald AM, Knight RC, Campbell MK, et al. What influences recruitment to randomised controlled trials? A review of trials funded by two UK funding agencies. Trials 2006;**7**(9).

43. Kaur G, Hutchison I, H M, et al. Barriers to recruitment for surgical trials in head and neck oncology: a survey of trial investigators. BMJ open 2013.

44. Wade J, Donovan J, Lane J, et al. It's not just what you say, it's also how you say it: opening the 'black box' of informed consent appointments in randomised controlled trials. Social science & medicine 2009;**68**(11):2018-28.

45. Braun V, Clarke V. Using Thematic Analysis in Psychology. Qualitative Research in Psychology 2006;**3**:77-101.

46. Joffe H, L. Y. Content and thematic analysis. In: Marks DF, L Y, eds. Research Methods for Clinical and Health Psychology. London: SAGE Publications Ltd, 2004.

47. Glaser BG, Strauss AL. *The Discovery of Grounded Theory*. Chicago: Aldine, 1967.

48. Charmay K. *Constructing grounded theory: a practical guide through qualitative analysis*. London: Sage, 2006.

49. Donovan J, Mills N, Smith M, et al. Quality improvement report: Improving design and conduct of randomised trials by embedding them in qualitative research: ProtecT (prostate testing for cancer and treatment) study. Commentary: presenting unbiased information to patients can be difficult. Bmj 2002;**325**(7367):766-70.

50. Realpe A, Adams A, Wall P, et al. A new simple six-step model to promote recruitment to RCTs was developed and successfully implemented. Journal of clinical epidemiology 2016;**76**:166-74.

For peer review only

Figure 1: Study Flow Diagram

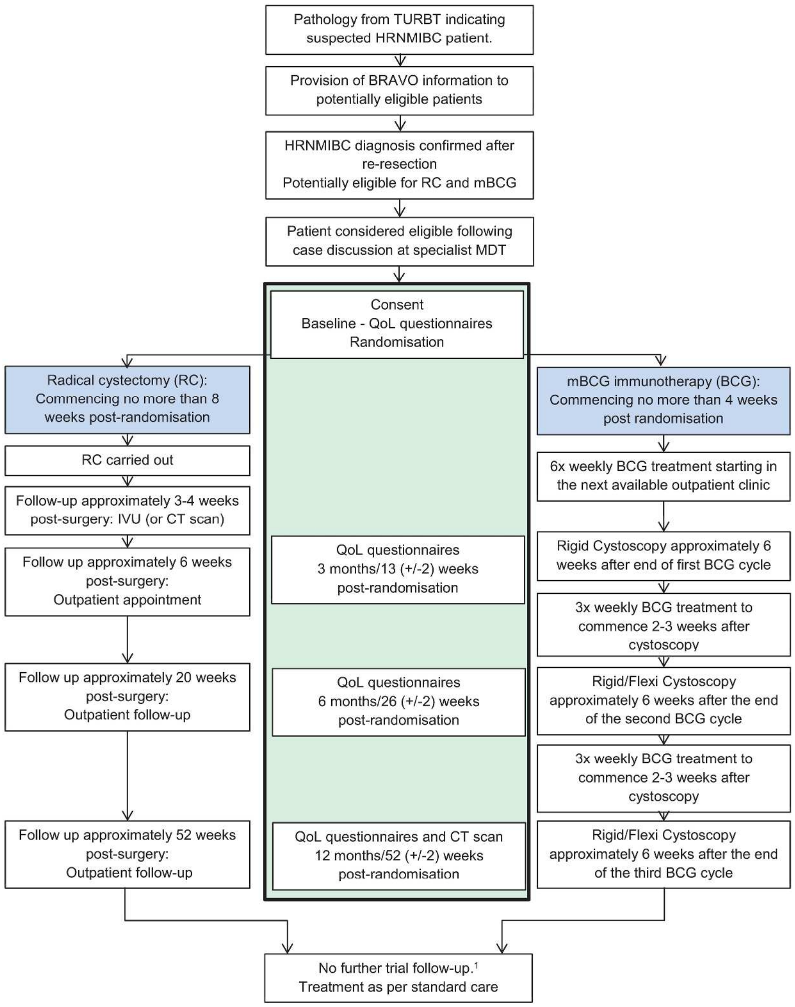

[1]Follow up will be limited to three months after the last participant has been randomised.

This is Figure 1

297x420mm (300 x 300 DPI)

Supplementary File 1

Qualitative Sub Study 1: Understanding patients' and health professionals' beliefs about BCG and radical cystectomy and potential barriers to recruitment.

Introduction

Lessons have been learned from previous large surgical randomised controlled trials (RCTs) (1), which suggest that when trials compare very different interventions, there are likely to be significant barriers to recruitment. A previous bladder cancer trial struggled to recruit (CRUK-SPARE trial), so this study comprises the preliminary work necessary for a feasibility RCT of mBCG versus primary radical cystectomy.

There are many barriers to recruitment and in the context of surgical trials we know that patient-factors, clinical-team factors, and information and consent related issues have all been identified as important considerations (2, 3). There will be a range of reasons for declining participation in the clinical trial including a lack of interest (4), not feeling well enough (5), fear of increased time commitments (4), and patient preferences (6). However, decisions not to participate may also be related to patients' misunderstandings regarding clinical trials (7) or how the healthcare professionals involved present the design and objectives of the study to the patient (7), and how the patient assimilates this information.

In the case of surgical trials, a need for staff training has been identified to ensure that both arms of the trial are presented in a balanced way so that patients understand the relative strengths and weaknesses of each, and there is also a recognised need for training about how to describe RCT methods (8). Radical cystectomy and BCG have been around in clinical practice for many years, so patients and health professionals may have a strong preference for either surgery or BCG and could feel that this choice is taken out of their hands by the randomization process. Understanding and addressing these issues will be crucial to the success of the feasibility trial whose aim will be to demonstrate that recruiting to a larger scale phase III trial is feasible.

It is therefore important that we have a clear understanding of patients' and health professionals' beliefs about these two treatments and ensure information presented to patients

For peer review only - http://bmjopen.bmj.com/site/about/guidelines.xhtml

by health professionals is done so in a way which minimises potential biases and facilitates an informed decision about participation. To address this, a tailored training package will be developed to enable staff to elicit and sensitively explore patient preferences for treatment, and facilitate an informed decision about participation. The development of the training package will be informed by existing evidence of what works (2, 9, 10) and content specific evidence derived from interviews with patients and healthcare staff to explore: a) treatment perceptions, b) barriers to participation, c) training needs of site staff.

Primary aims

- To understand patients' and professionals' beliefs about the two interventions and identify potential barriers to recruitment.
- To develop a training package for health professionals to aid informed decision making with patients

Secondary aims

- To elicit patients' beliefs and experiences of the two interventions (routes to diagnosis and beliefs about treatment options)
- To understand treatment burden and quality of life following treatment
- To elicit patient expectations of likely trial burden and barriers to participation
- To elicit patient recommendations for optimal recruitment and their views about randomisation
- To elicit health professional's beliefs about treatments, barriers to participation and perceived training needs

Outcome

Using the information gathered from the interviews and focus groups, and existing literature, develop a training package and associated materials and deliver the training package to staff to improve recruitment communication with patients.

For peer review only

Phase 1: ***Understanding Health Professionals views of bladder cancer treatment***

Design: Focus group study

Setting

Counselling and recruitment to the planned RCT will occur at the cancer referral centres, but patients are likely to discuss their treatment with the consultant at their local urological unit. To better understand the treatment beliefs of the health professionals (urologists, surgeons, nurses, research nurses, MDT co-ordinators and clinical nurse specialists) that patients may come in contact with, either to receive guidance on their treatment options, or to discuss the clinical trial, we approached staff from local units and referral centres. Packs were sent to the local Principal Investigator at each consenting site.

Inclusion Criteria

Staff involved in the recruitment of patients to the feasibility trial (MDT co-ordinators, surgeons, urologists, research nurses, clinical nurse specialists).

Sampling

We conducted focus groups with health professionals involved at different stages of the diagnosis pathway and trial recruitment pathway. A purposive sampling strategy was used to ensure we interviewed people involved across the diagnosis process, plus research nurses who would be involved in recruitment to the future trial. The sample included staff at local units and referral centres; nurses, (to include clincial nurse specialists and research nurses) (n=6-8), urologists and surgeons at local units and referral centres (n=6-8). We aimed to include senior and less experienced staff in each group.

Sample identification and consent process

All staff involved in the diagnosis process at each urological unit (local units and referral centres) were invited by letter to participate. An information pack (PIS, consent form, demographics form) was sent via the local Principal Investigator to their team.

For peer review only

Procedure

Two focus groups were undertaken (one each: nurses; clinicians); interviews (telephone or face to face) were offered to those who consented but could not attend the focus group. Focus groups were lead by an experienced qualitative researcher (MT) and supported by a second researcher. Written consent was taken at the beginning of the focus group. Discussions were informed by a topic guide which was informed by existing literature, (e.g. 9) clinical input and our PPI members, to include: beliefs about, and attitudes towards the interventions, barriers to recruitment, and training needs. The focus groups were audio-recorded with permission of the participants.

Data Analysis

Due to time and funding constraints, interviews were listened to and key sections transcribed for analysis. Personally identifiable data was removed or de-identified during transcription. The focus groups were analysed first, using an inductive, thematic coding approach. These were used to devise a coding frame for the interview transcripts. One researcher (JB) coded the remaining recordings, and a second researcher (MT) examined sections of data to check robustness of the themes.

Phase 2: Understanding patient views of bladder cancer treatments

Design: Semi-structured face-to-face interviews.

Inclusion criteria

- Aged 18 years or older
- Previous high grade (or grade 3) urothelial bladder cancer or non-muscle invasive tumour (diagnosed in previous 24 months – but not less than 4 months)
- Received either radical cystectomy or MBCG (or both)
- Able to provide written informed consent
- Able to converse in English (even if not first language)
- Currently or previously under the care of the urological units in Yorkshire and Humber.

For peer review only

Exclusion criteria

- Decline participation in the study
- Unable to comply with requirements of this protocol
- Unable to give informed consent

Study Setting

Participants were recruited from seven sites, to include patients treated at both local units and cancer referral centres.

Sampling

Due to the sensitive nature of bladder cancer, in-depth, semi-structured qualitative interviews were undertaken. We aimed for maximum variation in our sampling, with participants selected on the basis of socio-demographic factors (age, gender, experience of the intervention(s), geographic spread, and time since treatment). A sample of approximately 24 to 30 patients was expected.

Sample identification and consent process

Patients fitting the inclusion criteria were identified by the clinical team from clinic databases and an approach made in person, by telephone, or by post. Patients were also identified at regular clinic appointments and an information pack provided and verbal consent sought for the patient's details to be passed to the research team. At least 48 hours was given between being given the information pack and the phone call from the research team. If no response was received, a reminder letter was sent 14 days after the date of the first letter. If no response was received to the second request, no further contact was made.

When an approach was made by post, a pack containing a letter, demographics form, PIS, expression of interest form, consent form and freepost envelope was sent to the patient inviting them to participate. On return of the expression of interest (EoI) slip and demographics form, patients were contacted by the research team to discuss the study. Once consent has been received, patients were contacted to set up an appointment. For telephone interviews, a copy of the consent form was signed by the researcher and posted to the

For peer review only

participant. For face-to-face interviews, a copy of the signed consent form was given back to the participant on the day of the interview.

Patients were offered more time to consider participation and a number was provided that patients could use to contact the researcher. This recruitment strategy was selected because it minimises response bias and potentially increases the methodological rigour of the research (11).

Interview procedure

In depth semi-structured interviews were conducted with participants to elicit their beliefs about the two treatment options, their route to diagnosis, and to understand treatment burden and quality of life following treatment. A key role of the study was to understand and try to address issues around clinical trial participation, so we asked about likely trial burden, barriers to participation, recommendations for optimal recruitment and views about randomisation. Interviews were expected to last 45- 60 minutes. A topic guide was developed from the existing literature and discussions with the Chief Investigator, clinicians and Patient and Public Involvement members. Interviews were conducted by an experienced qualitative researcher. Since several studies (12, 13) show that there are no major differences in the results of telephone and face-to-face interviews, participants were given the option of a telephone interview to accommodate family and professional obligations. Interviews will be audio-recorded, with the permission of the participant.

Data analysis (as Phase 1 above)

Interviews were professionally transcribed verbatim and managed using NVivo. Personally identifiable data was removed or de-identified during transcription, and pseudonyms used. The data was analysed using Framework analysis (14) by three researchers independently coding the first three transcripts using initially inductive then deductive approaches. Codes and themes were compared after the analysis of the first three transcripts. Two researchers (AE & JB) then coded the remaining transcripts, with regular meetings with MT to ensure coding remains consistent. The analysis was further refined by using a constant comparison and contrastive approach, and looking for negative cases in order to examine for similarities and differences within and between patient groups.

Phase 3: Development of Training Package

The training package was developed from the findings of the interview and focus group data, and informed by the existing literature (9, 10). Training was delivered as a face-to-face workshop delivered at 3 sites and incorporated presentations and role play exercises with simulated patients (trained individuals who are regularly used in communication skills training throughout healthcare education) (15, 16). A manual was developed to accompany the training and included: detailed information about the trial and the two treatments, information on how to discuss uncertainty (of treatment options), how to describe randomisation, how to talk to patients who express a treatment preference. The aim of the training day was to allow staff to practice their communication skills in relation to the trial and receive feedback.

Results

The findings of the work are currently being written up for publication.

Ethical issues

Confidentiality

We were mindful of protecting participant confidentiality at all times. Audio recordings were stored on a secure drive and accessed only by the researcher team. After analysis the audio recordings were destroyed. Personally identifiable data was removed during transcription and pseudonyms adopted; these bear no resemblance to the patient's identity, hospital number, DOB or similar. Participants were asked to consent to direct quotes. Paper documents (e.g. consent forms, demographic questionnaires etc.) are kept in a secure office, and electronic information stored on University computers which are password protected. The file in which codes are linked to patients' names is stored on a password protected computer on a secure network. All data will be archived in accordance with University of Leeds and University of Sheffield NHS Foundation Trust procedures.

Informed consent

The patients were required to sign a consent form prior to getting involved to the sub-study. Those unable to consent for themselves were excluded from participating.

For peer review only

Time frame: October 2015 to September 2016.

Patient and Public Involvement

One lay member (PK), was involved in the development of the proposal. PK was involved in the design of the study, and has commented on the wording of this protocol, as well as the PIS, consent forms and topic guides used in this study. PK will remain involved in the study. A patient group was set up for the project and provided input into the study at key points in the project (study design, development of training manual, data analysis, and dissemination). Lay members participated in the training events to co-deliver the training package.

REC Review and reports

Approval for the study was sought and obtained (REF 15/LO/1864) and the study obtained R & D approvals from the NHS Trusts involved.

External Peer Review

This study is funded by Yorkshire Cancer Research and has undergone independent expert peer review, including review by a qualitative methodologist and a clinician.

For peer review only

References

1. Lane JA, Donovan JL, Davis M, Walsh E, Dedman D, Down L, et al. Active monitoring, radical prostatectomy, or radiotherapy for localised prostate cancer: study design and diagnostic and baseline results of the ProtecT randomised phase 3 trial. The Lancet Oncology. 2014;15(10):1109-18.
2. Moynihan C, Lewis R, Hall E, Jones E, Birtle A, Huddart R, et al. The Patient Deficit Model Overturned: a qualitative study of patients' perceptions of invitation to participate in a randomized controlled trial comparing selective bladder preservation against surgery in muscle invasive bladder cancer (SPARE, CRUK/07/011). Trials [Electronic Resource]. 2012;13:228.
3. Kaur G, Hutchinson I, Mehanna H, Williamson P, Shaw R, Tudur Smith C. Barriers to recruitment for surgical trials in head and neck oncology: a survey of trial investigators. BMJ Open [Internet]. 2013.
4. Biedrzycki BA, editor Decision making for cancer clinical trial participation: a systematic review. Oncology nursing forum; 2010.
5. NIHR-CRNCC. New from the Network [webpage]. 2011 [cited 2015]. Available from: http://www.crncc.nihr.ac.uk/Resources/NIHR%20CRN%20CC/Documents/NFTNW%20Issue%202%20Qtr%202%202010.pdf.
6. Mills EJ, Seely D, Rachlis B, Griffith L, Wu P, Wilson K, et al. Barriers to participation in clinical trials of cancer: a meta-analysis and systematic review of patient-reported factors. The Lancet Oncology. 2006;7(2):141-8.
7. Hussain-Gambles M, Leese B, Atkin K, Brown J, Mason S, Tovey P. Involving South Asian patients in clinical trials. 2004.
8. Fletcher B, Gheorghe A, Moore D, Wilson S, Damery S. Improving the recruitment activity of clinicians in randomised controlled trials: a systematic review. BMJ open. 2012;2(1):e000496.
9. Mills N, Blazeby J, Hamdy F, Neal DE, Campbell B, Wilson C, et al. Training recruiters to randomised trials to facilitate recruitment and informed consent by exploring patients' treatment preferences. Trials [Electronic Resource]. 2014;15(323).
10. Realpe A, Adams A, Wall P, Griffin D, Donovan JL. A new simple six-step model to promote recruitment to RCTs was developed and successfully implemented. Journal of clinical epidemiology. 2016;76:166-74.
11. Hewison J, Haines A. Confidentiality and consent in medical research: overcoming barriers to recruitment in health research. BMJ: British Medical Journal. 2006;333(7562):300.
12. Novick G. Is there a bias against telephone interviews in qualitative research? Research in nursing & health. 2008;31(4):391-8.
13. Sturges JE, Hanrahan KJ. Comparing telephone and face-to-face qualitative interviewing: a research note. Qualitative Research. 2004;4(1):107-18.
14. Ritchie J, Spencer L, O'Connor W. Carrying out qualitative analysis In: Ritchie J, Lewis J, editors. Qualitative Research Practice: A guide for social science students and researchers. London: Sage; 2003. p. 219-62.
15. Wykurz G, Kelly D. Developing the role of patients as teachers: a literature review. BMJ. 2002;325(818-21).
16. Bokken L, Rethans J, Scherpbier A, Van der Vieuten C. Strengths and weaknesses of simulated and real patients in the teaching of skills to medical students: a review. Simulation in Healthcare: The Journal of the Society of Medical Simulation. 2008;3:161-69.

For peer review only

SPIRIT 2013 Checklist: Recommended items to address in a clinical trial protocol and related documents*

Section/item	Item No	Description	Addressed on page number
**Administrative information**			
Title	1	Descriptive title identifying the study design, population, interventions, and, if applicable, trial acronym	1
Trial registration	2a	Trial identifier and registry name. If not yet registered, name of intended registry	4
	2b	All items from the World Health Organization Trial Registration Data Set	Yes, throughout.
Protocol version	3	Date and version identifier	37
Funding	4	Sources and types of financial, material, and other support	38
Roles and responsibilities	5a	Names, affiliations, and roles of protocol contributors	38
	5b	Name and contact information for the trial sponsor	38
	5c	Role of study sponsor and funders, if any, in study design; collection, management, analysis, and interpretation of data; writing of the report; and the decision to submit the report for publication, including whether they will have ultimate authority over any of these activities	38
	5d	Composition, roles, and responsibilities of the coordinating centre, steering committee, endpoint adjudication committee, data management team, and other individuals or groups overseeing the trial, if applicable (see Item 21a for data monitoring committee)	35

For peer review only

**Introduction**			
Background and rationale	6a	Description of research question and justification for undertaking the trial, including summary of relevant studies (published and unpublished) examining benefits and harms for each intervention	6-7
	6b	Explanation for choice of comparators	6-7
Objectives	7	Specific objectives or hypotheses	8
Trial design	8	Description of trial design including type of trial (eg, parallel group, crossover, factorial, single group), allocation ratio, and framework (eg, superiority, equivalence, noninferiority, exploratory)	7-8
**Methods: Participants, interventions, and outcomes**			
Study setting	9	Description of study settings (eg, community clinic, academic hospital) and list of countries where data will be collected. Reference to where list of study sites can be obtained	13-14
Eligibility criteria	10	Inclusion and exclusion criteria for participants. If applicable, eligibility criteria for study centres and individuals who will perform the interventions (eg, surgeons, psychotherapists)	9-11, 22-23
Interventions	11a	Interventions for each group with sufficient detail to allow replication, including how and when they will be administered	18-24
	11b	Criteria for discontinuing or modifying allocated interventions for a given trial participant (eg, drug dose change in response to harms, participant request, or improving/worsening disease)	18-24
	11c	Strategies to improve adherence to intervention protocols, and any procedures for monitoring adherence (eg, drug tablet return, laboratory tests)	26
	11d	Relevant concomitant care and interventions that are permitted or prohibited during the trial	18-24
Outcomes	12	Primary, secondary, and other outcomes, including the specific measurement variable (eg, systolic blood pressure), analysis metric (eg, change from baseline, final value, time to event), method of aggregation (eg, median, proportion), and time point for each outcome. Explanation of the clinical relevance of chosen efficacy and harm outcomes is strongly recommended	8-9, 27-28
Participant timeline	13	Time schedule of enrolment, interventions (including any run-ins and washouts), assessments, and visits for participants. A schematic diagram is highly recommended (see Figure)	Flow diagram, 25-27

For peer review only - http://bmjopen.bmj.com/site/about/guidelines.xhtml

Sample size	14	Estimated number of participants needed to achieve study objectives and how it was determined, including clinical and statistical assumptions supporting any sample size calculations	13
Recruitment	15	Strategies for achieving adequate participant enrolment to reach target sample size	31-32
**Methods: Assignment of interventions (for controlled trials)**			
Allocation:			
Sequence generation	16a	Method of generating the allocation sequence (eg, computer-generated random numbers), and list of any factors for stratification. To reduce predictability of a random sequence, details of any planned restriction (eg, blocking) should be provided in a separate document that is unavailable to those who enrol participants or assign interventions	17-18
Allocation concealment mechanism	16b	Mechanism of implementing the allocation sequence (eg, central telephone; sequentially numbered, opaque, sealed envelopes), describing any steps to conceal the sequence until interventions are assigned	17-18
Implementation	16c	Who will generate the allocation sequence, who will enrol participants, and who will assign participants to interventions	17-18
Blinding (masking)	17a	Who will be blinded after assignment to interventions (eg, trial participants, care providers, outcome assessors, data analysts), and how	8
	17b	If blinded, circumstances under which unblinding is permissible, and procedure for revealing a participant's allocated intervention during the trial	n/a
**Methods: Data collection, management, and analysis**			
Data collection methods	18a	Plans for assessment and collection of outcome, baseline, and other trial data, including any related processes to promote data quality (eg, duplicate measurements, training of assessors) and a description of study instruments (eg, questionnaires, laboratory tests) along with their reliability and validity, if known. Reference to where data collection forms can be found, if not in the protocol	25-27, 36
	18b	Plans to promote participant retention and complete follow-up, including list of any outcome data to be collected for participants who discontinue or deviate from intervention protocols	25-27

For peer review only - http://bmjopen.bmj.com/site/about/guidelines.xhtml

Data management	19	Plans for data entry, coding, security, and storage, including any related processes to promote data quality (eg, double data entry; range checks for data values). Reference to where details of data management procedures can be found, if not in the protocol	25-27
Statistical methods	20a	Statistical methods for analysing primary and secondary outcomes. Reference to where other details of the statistical analysis plan can be found, if not in the protocol	27-30
	20b	Methods for any additional analyses (eg, subgroup and adjusted analyses)	27-30
	20c	Definition of analysis population relating to protocol non-adherence (eg, as randomised analysis), and any statistical methods to handle missing data (eg, multiple imputation)	27-30
**Methods: Monitoring**			
Data monitoring	21a	Composition of data monitoring committee (DMC); summary of its role and reporting structure; statement of whether it is independent from the sponsor and competing interests; and reference to where further details about its charter can be found, if not in the protocol. Alternatively, an explanation of why a DMC is not needed	35
	21b	Description of any interim analyses and stopping guidelines, including who will have access to these interim results and make the final decision to terminate the trial	27-30
Harms	22	Plans for collecting, assessing, reporting, and managing solicited and spontaneously reported adverse events and other unintended effects of trial interventions or trial conduct	25-27
Auditing	23	Frequency and procedures for auditing trial conduct, if any, and whether the process will be independent from investigators and the sponsor	35
**Ethics and dissemination**			
Research ethics approval	24	Plans for seeking research ethics committee/institutional review board (REC/IRB) approval	35
Protocol amendments	25	Plans for communicating important protocol modifications (eg, changes to eligibility criteria, outcomes, analyses) to relevant parties (eg, investigators, REC/IRBs, trial participants, trial registries, journals, regulators)	38

For peer review only - http://bmjopen.bmj.com/site/about/guidelines.xhtml

Consent or assent	26a	Who will obtain informed consent or assent from potential trial participants or authorised surrogates, and how (see Item 32)	15-17
	26b	Additional consent provisions for collection and use of participant data and biological specimens in ancillary studies, if applicable	n/a
Confidentiality	27	How personal information about potential and enrolled participants will be collected, shared, and maintained in order to protect confidentiality before, during, and after the trial	33
Declaration of interests	28	Financial and other competing interests for principal investigators for the overall trial and each study site	35
Access to data	29	Statement of who will have access to the final trial dataset, and disclosure of contractual agreements that limit such access for investigators	37
Ancillary and post-trial care	30	Provisions, if any, for ancillary and post-trial care, and for compensation to those who suffer harm from trial participation	35
Dissemination policy	31a	Plans for investigators and sponsor to communicate trial results to participants, healthcare professionals, the public, and other relevant groups (eg, via publication, reporting in results databases, or other data sharing arrangements), including any publication restrictions	35, 37
	31b	Authorship eligibility guidelines and any intended use of professional writers	37
	31c	Plans, if any, for granting public access to the full protocol, participant-level dataset, and statistical code	36
**Appendices**			
Informed consent materials	32	Model consent form and other related documentation given to participants and authorised surrogates	36
Biological specimens	33	Plans for collection, laboratory evaluation, and storage of biological specimens for genetic or molecular analysis in the current trial and for future use in ancillary studies, if applicable	n/a

*It is strongly recommended that this checklist be read in conjunction with the SPIRIT 2013 Explanation & Elaboration for important clarification on the items. Amendments to the protocol should be tracked and dated. The SPIRIT checklist is copyrighted by the SPIRIT Group under the Creative Commons "Attribution-NonCommercial-NoDerivs 3.0 Unported" license.

For peer review only - http://bmjopen.bmj.com/site/about/guidelines.xhtml

Radical Cystectomy (Bladder Removal) against intra-vesical BCG immunotherapy for high risk non-muscle invasive bladder cancer (BRAVO): Protocol for a randomised controlled feasibility study

Journal:	*BMJ Open*
Manuscript ID	bmjopen-2017-017913.R1
Article Type:	Protocol
Date Submitted by the Author:	01-Jun-2017
Complete List of Authors:	Oughton, Jamie; University of Leeds, Leeds Institute of Clinical Trials Research<br>Poad, Heather; University of Leeds, Leeds Institute of Clinical Trials Research<br>Twiddy, Maureen; University of Leeds, Leeds Institute of Health Sciences<br>Collinson, Michelle; University of Leeds, Leeds Institute of Clinical Trials Research<br>Hiley, Victoria; University of Leeds, Leeds Institute of Clinical Trials Research<br>Gordon, Kathryn; University of Leeds, Leeds Institute of Clinical Trials Research<br>Johnson, Mark; Freeman Hospital<br>Jain, Sunjay; St James's University Hospital<br>Noon, Aidan; University of Sheffield Medical School, Academic Urology Unit<br>Chahal, Rohit; Bradford Teaching Hospitals NHS Foundation Trust<br>Simms, Matt; Hull and East Yorkshire Hospitals NHS Trust<br>Dooldeniya, Mohantha; Mid Yorkshire Hospitals NHS Trust<br>Koenig, Phillip; Airedale NHS Foundation Trust<br>Goodwin, Louise; University of Sheffield Medical School, Academic Urology Unit<br>Brown, Julia; University of Leeds, Leeds Institute of Clinical Trials Research<br>Catto, James; University of Sheffield Medical School, Academic Urology Unit
**Primary Subject Heading**:	Oncology
Secondary Subject Heading:	Surgery, Urology
Keywords:	High risk non muscle invasive bladder cancer, HRNMIBC, mBCG, radical cystectomy, feasibility study, bladder cancer

SCHOLARONE™
Manuscripts

For peer review only - http://bmjopen.bmj.com/site/about/guidelines.xhtml

Radical Cystectomy (Bladder Removal) against intra-vesical BCG immunotherapy for high risk non-muscle invasive bladder cancer (BRAVO): Protocol for a randomised controlled feasibility study

For peer review only

Jamie B Oughton[1], Heather Poad[1], Maureen Twiddy[2], Michelle Collinson[1], Victoria Hiley[1], Kathryn Gordon[1], Mark Johnson[4], Sunjay Jain[5], Aidan P. Noon[3], Rohit Chahal[6], Matt Simms[7], Mohantha Dooldeniya[8], Phillip Koenig[9], Louise Goodwin[3], Julia M. Brown[1] and James W.F. Catto[3] for the BRAVO study group*

[1]Clinical Trials Research Unit, Leeds Institute of Clinical Trials Research, University of Leeds, Leeds, LS2 9JT, UK; [2]Leeds Institute of Health Sciences, University of Leeds, Leeds, UK; [3]Academic Urology Unit, University of Sheffield, Sheffield, UK; [4]Freeman Hospital, Newcastle, UK; [5]St James's University Hospital, Leeds, UK; [6]Bradford Teaching Hospitals NHS Foundation Trust, Bradford, UK; [7]Hull and East Yorkshire NHS Trust, Hull, UK; [8]Mid Yorkshire Hospitals NHS Trust, Wakefield, UK; [9]Airedale NHS Foundation Trust, Keighley, UK.

Corresponding Author:

Correspondence to;

James Catto, Academic Urology Unit, G Floor, The Medical School,

University of Sheffield, Beech Hill Road, Sheffield, S10 2RX, United Kingdom

Tel: +44 (0)114 226 1229

Fax: +44 (0)114 271 2268

Email: j.catto@sheffield.ac.uk

Word count: 5370

* BRAVO study group members: Zahir Abbasi, Linzi Bone, Angela Brocklehurst, Nicolas Bryan, Lindsay Caygill, Russsell Dowde, Jan Farrell, Jonathan Gill, Lisa Gledhill, Susan Hill, Helen Hothersall, Anne Kay, Phil Kelly, Bernadette Kilbane, Sanjeev Kotwal, Kate

For peer review only - http://bmjopen.bmj.com/site/about/guidelines.xhtml

Linton, Stephen Littler, Tiago Mendonca, Suzanne Miles, Stephen Mitchell, Peter Murphy, Vicky Murray, Naledi Mzwimbi, Alan Paul, Ramanan Rajasundaram, Julie Rawlings, Hannah Roberts, Helen Robertshaw, Wendy Robson, Derek Rosario, Kully Sandhu, Paul Sagar, Alison Shaw, Rajindrah Singh, Nick Smith, Tina Soar, Zoe Storton, Beverley Taylor, Francis Thomas, Nicky Thomas, Suresh Venugopal, Rachel Walker, Louise Weatherley, and Susan Wormley. Independent steering group members: Alison Birtle (Chair), Simon Fulford and Chris Metcalfe.

For peer review only

For peer review only - http://bmjopen.bmj.com/site/about/guidelines.xhtml

ABSTRACT

Introduction: High risk non-muscle invasive bladder cancer (HRNMIBC) is a heterogeneous disease which can be difficult to predict. Whilst around 25% of cancers progress to invasion and metastases, the remaining majority of tumours remain within the bladder. It is uncertain whether patients with HRNMIBC are better treated with intravesical maintenance BCG (mBCG) immunotherapy or primary radical cystectomy (RC). A definitive randomised controlled trial (RCT) is needed to compare these two different treatments, but may be difficult to recruit to and has not been attempted to date. Before undertaking such an RCT it is important to understand whether such a comparison is possible and how best to achieve it.

Methods and analysis: BRAVO is a multi-centre, parallel-group, mixed-methods, individually randomised, controlled, feasibility study for patients with HRNMIBC. Participants will be randomised to receive either mBCG immunotherapy or RC. The primary objective is to assess the feasibility and acceptability of performing the definitive phase III trial via estimation of eligibility and recruitment rates, assessing uptake of allocated treatment and compliance with mBCG, determining quality of life questionnaire completion rates and exploring reasons expressed by patients for declining recruitment into the study. We aim to recruit 60 participants from 6 centres in the UK. Surgical trials with disparate treatment options find recruitment challenging from both the patient and clinician perspective. By building on the experiences of other similar trials through implementing a comprehensive training package aimed at clinicians to address these challenges (qualitative sub study), we hope that we can demonstrate that a phase III trial is feasible.

For peer review only - http://bmjopen.bmj.com/site/about/guidelines.xhtml

Ethics and dissemination: The study has ethical approval (16/YH/0268). Findings will be made available to patients, clinicians, the funders, and the NHS through traditional publishing and social media.

Trial Registration: ISRCTN12509361 Registered 06/09/16.

Keywords: High risk non-muscle invasive bladder cancer, BCG, radical cystectomy, feasibility study, bladder cancer, surgical trial, RCT

For peer review only

Strengths and limitations of this study

- This is an important comparison that has not been attempted before.
- This study will not determine which intervention is the superior treatment, a definitive phase III trial will still be needed.
- Recruitment may be challenging and may not be possible through traditional care pathways.

For peer review only

For peer review only - http://bmjopen.bmj.com/site/about/guidelines.xhtml

INTRODUCTION

Context

Bladder cancer (BC) is a common disease that is one of the most expensive malignancies to manage[1]. Around 25% of patients present with poorly differentiated, low stage tumours; termed 'high risk non-muscle invasive bladder cancer' (HRNMIBC; including tumours with carcinoma in situ, invasion into the lamina propria and intra-epithelial spread into the prostatic urethra). The two main treatment options for HRNMIBC are intra-vesical immunotherapy (using a maintenance regime of intravesical *bacillus Calmette-Guerin* (mBCG)) and radical cystectomy (RC). The former aims to induce an immune response against the tumour and may reduce the risk of progression to muscle invasion[2]. Whilst mBCG avoids bladder removal, it leaves patients at risk of local progression and may impact upon quality of life (QoL) through local symptoms and anxiety. RC removes the risk of local disease progression and may have the best oncological outcomes, but could be overtreatment for non-progressing tumours. Many patients develop short-term post-operative complications after RC and others have a reduction in QoL following surgery. To date, RC and mBCG have not been directly compared. Their comparative risks and benefits are unknown, hampering decision-making, clinical care and exposing patients to both over and under-treatment.

Current knowledge

The natural history of HRNMIBC is unpredictable. Rates of progression to muscle invasion and metastases vary between 25-75%[3] and long term outcomes suggest around 20-25% of patients with HRNMIBC may die from BC[4 5]. mBCG avoids bladder removal and meta-analyses report potential reductions in progression by 5% at 2.5 years[6]. However, mBCG can be poorly tolerated, its impact upon progression is debated[2] and there are manufacturing

For peer review only - http://bmjopen.bmj.com/site/about/guidelines.xhtml

problems[7]. mBCG involves 27 intravesical instillations and 10 cystoscopies over 3 years. Many (74%) patients report local and systemic toxicity[8 9], so only 30% of patients complete mBCG[9 10]. Furthermore, there are few data to support that mBCG with bladder preservation preserves a good quality of life (QoL). With regards to oncological outcomes, reports of BC's-failing mBCG find upstaging to invasion in 27-63% of tumours and the cancer specific survival is worse than for BC with *de novo* muscle invasion (e.g. 37% vs 67%/3 years)[11-15]. RC includes removal of the bladder and adjacent organs, and reconstruction of urinary drainage. Many patients develop short-term bowel, respiratory or cardiovascular problems, including up to 20% require intervention[16]. Prospective studies report recovery of QoL following RC takes 6 months or longer to recover to pre-operative levels[17]. Recurrence-free survival rates following primary RC for HRNMIBC cancers appear superior to those from mBCG (e.g. 79%/10-years)[18].

Surgical RCTs

As contemporary data challenge the role of mBCG[2] and lessons have been learnt from large surgical RCTs[19], we believe it is time to compare mBCG with RC. This is an important comparison and this opportunity may be lost as RC for HRNMIBC becomes more popular[20]. Importantly, the 2015 NICE Bladder cancer guidelines selected this comparison as one of the highest ranked research priorities in the disease[21]. The BRAVO study aims to compare surgical and non-surgical treatments. Trials of similarly disparate treatments in BC have previously failed to recruit (e.g. CRUK-SPARE trial)[22 23]. Here we propose the preliminary work necessary to understand if we can undertake a large RCT of mBCG versus RC. Anticipated barriers to recruitment include patient and clinician-preferences, BC treatment pathways, a lack of high quality information[24 25], and the need for staff training in equipoise

For peer review only - http://bmjopen.bmj.com/site/about/guidelines.xhtml

and communicating RCT methods[26]. To address these issues, we will develop a tailored staff training package to facilitate informed decision making about participation and to better understand RCT methodology. The development work will be informed by existing knowledge[24 27 28] and context-specific evidence derived from interviews with patients and healthcare staff exploring: a) treatment perceptions, b) patient pathways to treatment; c) barriers to participation, d) training needs of site staff. This qualitative work to develop and deliver the training package is described in a separate protocol (Supplementary File 1). We will then undertake a feasibility study to assess whether recruitment could be achieved in a definitive trial, embedding a qualitative component to establish patient experience.

Study Aims

Our aims are to assess whether a larger phase III RCT is possible and to acquire sufficient data to aid planning such a trial. Primary outcomes are:

1. To assess the number of patients screened and identified as eligible within these 6 centres.
2. To assess recruitment rates (number of patients randomised per month).

Secondary outcomes are:

1. To assess acceptance of allocated treatment.
2. To assess the rate of compliance with mBCG at 12 months after randomisation and collect reasons for non-compliance.
3. To assess the feasibility and optimal frequency of collecting QoL data in patients treated for HRNMIBC.
4. To obtain preliminary data on the QoL data of patients treated for HRNMIBC.
5. To explore the reasons expressed by patients for declining recruitment into the study.

For peer review only - http://bmjopen.bmj.com/site/about/guidelines.xhtml

METHODS AND DESIGN

Trial Design

BRAVO is a multi-centre, parallel-group, mixed-methods, individually randomised, controlled feasibility study in patients with HRNMIBC suitable for treatment by either mBCG or RC. Eligible, consenting patients will be randomised (1:1) to receive either mBCG or RC (Figure 1). Due to the different treatment modalities in the two arms, it is not feasible to blind patients or clinicians to treatment allocation. Patient reported outcome data will be collected at 3, 6 and 12-months post-randomisation in clinic or by postal questionnaire if the patient is not due to attend a clinic visit.

Trial Population

We aim to recruit 60 patients from 6 UK cancer centres and their associated District General Hospitals. The inclusion criteria are:

1. Male or female aged ≥ 18 years old.
2. Patients with a new diagnosis of high-risk (high grade[29] or grade 3[30]) non-muscle invasive urothelial carcinoma (staged as either pTa, pTis or pT1). Patients with previous low grade non muscle invasive bladder cancer (NMIBC) are eligible.
3. The tumour is either solely urothelial cell carcinoma or has urothelial cell carcinoma as the majority histological component.
4. In addition to the HRNMIBC bladder tumour, there needs to be one or more risk factor from:
   a. Presence of pTis in the bladder
   b. Presence of pTis in the prostatic urethra
   c. Lymphovascular invasion

For peer review only

   d. Vascular invasion
   e. Residual Grade 3/High grade UCC on re-resection (or initial TURBT if no re-resection)
   f. Multifocal disease (>3 tumours at initial resection)
   g. Young age (<65 years old)
   h. Initial tumour Size > 3cm (or >5g in histology specimen)
   i. pT1 stage

5. *Either* re-resection of the bladder (following the initial diagnostic TURBT) within the 3 months prior to randomisation confirming the absence of muscle invasion

   OR

   a. the initial diagnostic TURBT biopsy contains muscle, AND
   b. the radiological and pathological stage assessment are in agreement regarding stage and absence of muscle invasion, AND
   c. a re-resection is not appropriate in the opinion of the treating clinician AND
   d. the initial TURBT is within 3 months prior to randomisation.

6. CT or cross sectional imaging of the abdomen and pelvis within the year prior to starting treatment.
7. Imaging of the lungs and thorax within 3 months prior to randomisation.
8. Suitable and fit for both mBCG and RC as determined by the treating clinician.
9. Central MDT pathological review agrees diagnosis.
10. If female, must be (as documented in patient notes):

    a. postmenopausal (no menses for 12 months without an alternative medical cause), or

For peer review only

For peer review only - http://bmjopen.bmj.com/site/about/guidelines.xhtml

b. surgically sterile (hysterectomy, bilateral salpingectomy or bilateral oophorectomy), or

c. using acceptable contraception (which must be continued for 7 days after the last dose of BCG or until RC is carried out). Women of child bearing potential must undergo a pregnancy test before randomisation.

d. not breast feeding.

The exclusion criteria are:

1. Solely non-urothelial or any variant urothelial pathology
2. Unable or not willing to give informed consent
3. Previous high risk (high grade or grade 3) NMI or invasive bladder cancer
4. Any previous treatment with intravesical BCG
5. Any previous treatment with pelvic radiotherapy
6. Any other malignancy (excluding non-melanomatous skin cancer, low-risk prostate cancer and prior low risk bladder cancer)

Eligibility waivers are not permitted.

Prior to entry, patients must be accurately staged (e.g. cross sectional imaging (e.g. CT) of the abdomen, pelvis and thorax, or bone scan if indicated, within 3 months prior to randomisation) and judged to be eligible for both treatments (anesthetic evaluation in those with borderline fitness for RC). After trial entry, women of childbearing age must be proven to be not pregnant (pregnancy test).

Sample Size

For peer review only

The sample size for this feasibility study has been set to give confidence that the recruitment target for the main trial can be met. A formal power calculation is not appropriate as effectiveness is not being evaluated. It is estimated that per year, over the six centres there will be approximately 1000 new diagnoses of NMIBC, where 20% are likely to be eligible (200 patients)[31]. We would need to show that we are able to randomise approximately 25% of all eligible patients to be confident that the recruitment target for the main trial would be met within 3 years, with an additional 9 centres. We therefore plan to recruit 60 patients over an 18 month period in the feasibility study. For the phase III trial, we anticipate either a single primary endpoint (cancer-specific survival) or co-primary endpoints (cancer-specific survival and averaged QALYs). We estimate 506 participants are required to have 80% power to show a superiority hazard ratio of 0.626 (based on an improvement in 5-year cancer specific survival from 70% in the BCG arm to 80% in the RC arm), assuming a 3-year accrual period, 5 years of follow-up, and accounting for 5% loss-to-follow-up.

Setting

Participants will be recruited from 6 cancer centres (and 7 neighbouring district hospitals) within Yorkshire and Northumberland. NHS demographic data show that Yorkshire and Northumberland have some of the highest rates of BC incidence and some of the lowest rates of survival from this cancer[32 33].

Recruitment

Patients will be identified through multidisciplinary team (MDT) meetings and approached once they know their diagnosis of HRNMIBC. This approach may be at any hospital involved in their care and by medical or nursing staff. The team will introduce the trial when treatment options are being discussed, provide the introduction leaflet and ask permission

For peer review only - http://bmjopen.bmj.com/site/about/guidelines.xhtml

(and contact details) for a Research Nurse to contact the patient with more information. The number of eligible and screened patients will be recorded. Interested participants will be invited to attend an appointment at the research site and/or receive telephone calls, to be given a full explanation of the BRAVO study. Experience in similar studies suggests patients can be overwhelmed by information given in clinic, and that telephone contact can help and provides another opportunity to support patients. Up to five attempts will be made to contact the participant by telephone, after which it will be assumed they have decided to not participate. Eligible patients can be contacted by post if the immediate care team deem this best. No contact information will be shared outside of the team directly caring for the patient unless consent has been obtained.

Consent

Informed consent takes place in a face to face setting at the research site. Patients will have at least 24 hours to consider participation and will be encouraged to discuss the study with their family and other healthcare professionals. A full verbal explanation of the study, a written PIS (Patient Information Sheet detailing rationale, design and personal implications of trial entry) and informed consent form will be provided. Participants may withdraw at any stage of the trial. Consent will be obtained prior to collection of baseline assessment data and subsequent randomisation.

Staff training

We recognise the challenge of comparing these two treatment choices and that the patient pathway includes interaction with numerous healthcare providers. To minimise bias and to maintain equipoise, a training package will be developed from interviews with patients and clinicians and delivered to staff who are likely to care for patients before and during the

For peer review only - http://bmjopen.bmj.com/site/about/guidelines.xhtml

study. Training will incorporate lectures and role play exercises with simulated patients. A careful explanation of the potential risks and benefits of the two treatment interventions is crucial, such risks will be clearly explained to interested patients in an unbiased and fair way, assisted by written study-specific patient information.

Randomisation

Patients will be randomised, using a 24-hour centralised telephone or web based randomisation system, on a 1:1 basis to receive either RC or mBCG. A computer-generated adaptive minimisation algorithm that incorporates a random element will be used to ensure the treatment groups are balanced (stratified) for:

- Age (<75, >=75)
- Sex (male, female)
- Recruiting cancer centre
- Tumour stage (pTa/pTis, pT1)
- Presence of carcinoma in situ (Yes, No)
- Previous low risk bladder cancer (Yes, No)

Intervention - BCG immunotherapy

Maintenance BCG immunotherapy will be administered at either the cancer centre or district general hospital using the SWOG protocol[10]. At least 12 months of BCG treatment are required and 6 weeks of induction BCG will be followed by 3 doses at 4 and 10 months after diagnosis. Delays and deferrals are common and allowed within this study. BCG induction should include at least 4 (of 6) doses of BCG and induction should be completed within 10 weeks. The presence of an invasive BC requires the cessation of mBCG and a change in

For peer review only - http://bmjopen.bmj.com/site/about/guidelines.xhtml

For peer review only

treatment intent. Maintenance BCG may continue in the presence of low risk NMI and HRNMI bladder cancer at the first cystoscopy, thereafter these are managed as recurrences and require patient discussion. Rigid cystoscopy with bladder biopsy and bladder washings is mandated at the first check. After this, bladder surveillance is performed as per local protocol (flexible or rigid instruments). All cystoscopies will be undertaken or directly supervised (with a visual check) by a Consultant Urologist who manages HRNMIBC. Fluorescence or narrow band imaging may be used, as per local protocols. Histological review of the bladder biopsies and urinary cells should be performed to determine the presence or absence of BC.

Local and systemic complications are common in mBCG regimens and should be managed as per local protocol. The study will collect data on the frequency of expected BCG toxicities and whether this leads to the cessation of BCG treatment. Cystectomy may be performed within BRAVO for severe BCG-related toxicities, if these warrant such an intervention. Patients undergoing BCG treatment may stop treatment due to disease progression, disease recurrence, serious BCG intolerance or side effects or patient choice. Disease progression: patients who have confirmed progressive disease after any of the check cystoscopies (presence of pT2 tumours, cancer in lymph nodes, or metastases) should stop BCG and be offered curative treatment for muscle invasive bladder cancer. Disease recurrence is defined as the presence of low risk NMI or HRNMIBC from the second check cystoscopy onwards. Participants with recurrence should be offered the option of changing treatment, including radical cystectomy or using second line intravesical approaches.

Intervention - Radical Cystectomy

Radical cystectomy should be performed at each cancer centre by teams specialising in this service. Variations in surgical performance and practice produce wide differences in

For peer review only

morbidity and mortality from RC[34]. To mitigate these, surgeons within BRAVO will have individually undertaken at least 10 RCs per year for the last 2 years (or 20 in the last year), have median length of stay rates under 16 days, have 90-day post-RC mortality rate of less than 10% (collected outcomes from the British Association of Urological Surgeons (BAUS) RC complex dataset[31]). Post-operative complication rates and intra and post-operative transfusion rates will also be taken into consideration. Individual surgeon data will act as surrogate measures for the entire surgical team and require accreditation from the Trial Management Group before entry into BRAVO. Submitted data for surgical accreditation should reflect the practice to be undertaken within this study (e.g. open or robotic approaches). Surgery should take place within 8 weeks of randomisation.

Cystectomy should include removal of adjacent organs. In males, this includes the prostate and seminal vesicles. In females, this should include a section of adjacent anterior vaginal wall, the uterus, cervix and fallopian tubes and, if no bladder reconstruction is planned, the urethra. Oophorectomy is optional, as per local practice and individualised for each patient. Pelvic lymphadenectomy is mandated within BRAVO. The template should at least include the regional lymph nodes up to the level of the ureteric crossing of the common iliac vessels. This includes the obturator fossa, the external iliac and internal iliac nodes. A more extended lymphadenectomy is acceptable. Excised lymphatic tissue should be submitted for histological analysis. Perioperative care is to be carried out as per Enhanced Recovery After Surgery (ERAS) protocols[35 36].

Withdrawal of treatment

In line with usual clinical care, cessation or alteration of regimens will be at the discretion of attending clinicians or the participants. All participants who withdraw or are withdrawn from

For peer review only

For peer review only - http://bmjopen.bmj.com/site/about/guidelines.xhtml

their allocated treatment will still attend for follow-up assessments and complete questionnaires unless unwilling to do so and outcomes will continue to be collected. In the event that a patient withdraws consent prior to randomisation, data collected up to the point of withdrawal will be analysed.

Data collection

A screening form, to include demographic details and reasons for ineligibility, exclusion or refusal, will be completed for all patients considered for BRAVO. A feedback questionnaire will be used to identify patients who are willing to take part in the qualitative sub study (Supplementary File 1). Baseline assessments prior to randomisation include QoL scores (EuroQuol-5D (EQ-5D) [37], EORTC QLQ-C30 [38], EORTC QLQ-BLM30) at trial entry.

Within mBCG, outcomes and compliance data will be collected at each cystoscopy. For RC, patient and operative data will be collected at the time of surgery, as per our national register [31], and then at each subsequent follow up visit (3, 6 and 12 months post randomisation). Follow up imaging (CT scan) to assess response to treatment will be performed in both arms at one year post randomisation. QoL questionnaires will be collected at 3, 6 and 12 months post-randomisation in face to face consultations or by telephone. These include EuroQuol-5D (EQ-5D) [37], EORTC QLQ-C30 [38], and either EORTC QLQ-BLM30 (for those randomised to RC) or EORTC QLQ-NMIBC24 (for those randomised to BCG). Information will be collected on deaths, complications and toxicities (adverse events), and related and unexpected serious adverse events up to one year post randomisation, or three months after the last participant is randomised if earlier.

For peer review only - http://bmjopen.bmj.com/site/about/guidelines.xhtml

Statistical analyses

A detailed statistical analysis plan will be written before any analysis is undertaken. All analyses and data summaries will be conducted on the intention-to-treat (ITT) population. No formal interim analyses are planned and final analysis will take place when all available data have been received. The analysis will focus on descriptive statistics and confidence interval estimation. Primary analysis will include summaries of the number of patients at each stage of the recruitment pathway (screening, eligibility, consent and randomisation) and assessment of the overall monthly recruitment rate. Secondary analysis will include summaries of acceptance of randomised treatment and mBCG treatment compliance. Participant retention and self-reported QoL outcomes during follow-up, including withdrawal data (timing and reason), will also be summarised overall and by time-point. Levels of missing data in QoL outcomes will be assessed. The median cancer-specific survival estimate and its corresponding 95% confidence interval (CI) will be calculated to inform the sample size calculation of the phase III trial. As this is to aid the design of a pragmatic phase III trial, all randomised patients will be included in the calculation, regardless of treatment received. Cancer-specific survival will be calculated from the date of randomisation to the date of cancer-specific death. Participants with missing follow-up data, or who are alive at the time of the analysis will be censored at the date they were last known to be alive. Overall survival, calculated from the date of randomisation to the date of death, will also be summarised as for cancer-specific survival.

The frequent collection of QoL data within this feasibility study is necessary in order to assess the burden to patients. This will be assessed by monitoring collection compliance rates and will inform the optimal frequency of data collection for the main trial. Averaged QALYs

For peer review only - http://bmjopen.bmj.com/site/about/guidelines.xhtml

may be a co-primary endpoint for the main trial, as such, determining the optimal frequency of EQ-5D data collection within this feasibility study is crucial.

Safety

The number of adverse events and related unexpected serious adverse events will be summarised descriptively by arm, by grade, and body system. The proportion of participants experiencing each toxicity will be summarised by maximum NCI CTCAE grade[39] experienced, overall and by arm. Operative RC complications will be graded using the Clavien Dindo classification[40].

Criteria for progression to the definitive phase III trial

The following guidelines for progression to a definitive phase III trial have been defined:

- The recruitment and follow-up rates must demonstrate that a definitive trial using similar procedures will achieve sufficient power to test the hypothesised difference between treatment arms.
- The sample size calculation for the feasibility study and proposed phase III trial are provided earlier. This assumes that 20% of all new diagnoses of NMIBC would be eligible and approximately 25% of those would be randomised. To proceed to a definitive trial, we need to show that at least 20% of eligible patients can be randomised.

Qualitative sub study

There are two qualitative studies. The first was undertaken prior to the start of the RCT to identify a priori the barriers to recruitment from the perspectives of patients and staff to inform the development of a bespoke training package for staff[41] (see Supplementary File 1). A second qualitative study is embedded into the RCT trial to understand patients' views and

For peer review only

For peer review only - http://bmjopen.bmj.com/site/about/guidelines.xhtml

experiences of the treatments and explore patients' acceptability of the study and recruitment processes:

Qualitative sub study objectives

1. To gauge patients understanding of the study and their views on the recruitment process.
2. To qualitatively explore patient's acceptability of the study to assist in optimisation of recruitment strategies employed for the definitive trial.
3. Explore reasons for participation and non-participation of eligible patients.
4. Understand patients' experience of the randomisation process on decision making.
5. Understand why people refuse to participate or do not take up allocated treatment.
6. Patient understanding of study materials i.e. do patients understand what will happen if they take part and do they understand what they are being randomised to.
7. Acceptability of study procedures.
8. Acceptability of randomisation.

Qualitative sub study overview

In order to examine the views and experiences of bladder cancer patients we will conduct in-depth semi-structured interviews with patients approached to take part in the trial. Qualitative findings will help illuminate the acceptability of trial processes and explore barriers to uptake.

Recruitment to RCTs with very different treatment arms can be difficult and recruitment to trials involving surgery is particularly challenging[42]. Trials present practical and methodological challenges, including difficulties in recruitment, randomisation and lack of clinical equipoise[43]. Understanding why patients do or do not participate in trials is important

For peer review only - http://bmjopen.bmj.com/site/about/guidelines.xhtml

and clinical trials have recently begun to incorporate a qualitative component to address these issues. These studies have been able to successfully identify aspects of the trial design that hindered recruitment and identify possible solutions[42 44].

Qualitative sub study design

All eligible patients will be asked to complete a questionnaire to gauge their understanding of BRAVO and their views on the recruitment process. We will collect data from patients who decline the study, who consent but refuse allocation and those who consent and accept allocation. A short questionnaire will be given to seek patient views on the recruitment process and to ask if participants would be willing to provide detailed feedback by face to face or telephone interview. A purposive sample of 15 patients will be selected for interview. Written consent will be taken prior to the interview and a flexible topic guide developed in conjunction with PPI representatives, clinical colleagues and informed by the literature used to assist questionning. The topic guide will be devised to ensure the key issues are covered but do not dictate data collection; and will be flexible enough to elicit participants own experiences and views of the trial as well as issues unanticipated by the interview team. Interviews will be audio-recorded, transcribed and anonymised to protect confidentiality. With their consent, participants may be contacted after the interview to answer questions which may emerge during the analysis, or to explore issues that emerged in the interviews in more depth.

Qualitative sub study data analysis

Qualitative data will be analysed by the qualitative researcher. Interview transcripts will be checked for accuracy and then managed using NVivo qualitative data analysis software (QSR International, Daresbury, UK) which aids the indexing of qualitative data. Analysis will start during data collection and will inform later data collection; for example emerging themes may

For peer review only - http://bmjopen.bmj.com/site/about/guidelines.xhtml

identify new questions to explore in later interviews. The data will be analysed using thematic analysis[45 46] using an inductive (bottom-up) approach to identify and analyse patterns across the data set using constant comparison methods[47 48]. Inductive coding will follow using a line-by-line coding approach, with codes assigned to segments of data which provide insight into participants' views of the trial. An initial coding frame will be developed from the first interviews and will be modified, if necessary, as the analysis develops. A subset of transcripts will be independently coded by another member of the team and compared to ensure consistency. Any discrepancies will be discussed with the research team and resolved to achieve coding consensus. The data will be examined for negative cases and the reasons explored by comparison with the overall dataset.

Data Monitoring

Trial supervision includes a core project team, a trial management group (TMG), and an independent Trial Steering Committee (TSC). For a feasibility study of this nature and duration, a separate data monitoring and ethics committee is not required; rather; the TSC adopts a safety monitoring role and will review safety issues if this becomes necessary. Data will be monitored for quality and completeness by the CTRU. Missing data (except individual items collected via questionnaires) will be chased until received, confirmed as not available or the trial is at analysis. Any protocol changes will be disseminated by the CTRU to the relevant parties.

Trial Organisation and Administration

The trial was developed by the BRAVO Trial Management Group (TMG). The trial is funded by Yorkshire Cancer Research and is sponsored by the Sheffield Teaching Hospitals NHS Trust (Clinical Research Office, Royal Hallamshire Hospital, D Floor, Glossop Road,

For peer review only - http://bmjopen.bmj.com/site/about/guidelines.xhtml

Sheffield), co-ordinated by the CTRU, University of Leeds, and is registered (ISRCTN12509361). The trial will be conducted in accordance with the principles of Good Clinical Practice in clinical trials, as applicable under UK regulations, the NHS Research Governance Framework and through adherence to CTRU standard operating procedures (SOPs). CTRU/sponsor have systems in place to ensure that serious breaches of GCP or the trial protocol are identified and reported. Ethical approval has been obtained from the National Research Ethics Service Committee Yorkshire & Humber – South Yorkshire (reference 16/YH/0268). Any on-site source data verification carried out by the CTRU is not independent from sponsor. Sheffield Teaching Hospitals NHS Trust will not be liable for negligent harm caused by the design of the trial. No additional compensation for clinical negligence will be provided for trial participants over that which is available to NHS patients. All identifiable information collected during the course of the study will be kept strictly confidential and not transferred outside of the research team. Patient name (via consent form), email address and telephone number will be collected when a patient is randomised into the study but all other data collection forms that are transferred to or from the CTRU will be coded with a study number and will include two patient identifiers, usually the patient's initials and date of birth. Both electronic and paper data will be held in a secure locations with restricted access.

DISCUSSION

The 2015 NICE bladder cancer guidelines identified the comparison between mBCG and RC as one of their highest research priorities[21]. This reflects the importance of this question, but does not address how randomisation between two very different treatment options should occur or whether such a comparison is possible. Within this feasibility study we are

For peer review only - http://bmjopen.bmj.com/site/about/guidelines.xhtml

attempting to understand, address and develop methodology to allow such a comparison. This will require several key issues to be addressed. Firstly, it is clear from other surgical vs. non-surgical treatment trials[49] that the most important element for RCT recruitment is keeping equipoise when discussing the treatment options by medical and nursing staff. Whilst previous studies used research nurses to keep equipoise, this is not viable across many centres within the current research funding climate. In an attempt to replicate this model we ran a number of educational days to train relevant medical and nursing staff about the importance of equipoise and to discuss their beliefs about HRNMIBC. All staff had opinions about the efficacy of BCG and the quality of life with RC, and so it was important to discuss these in an open forum to challenge these views and use evidence to dispel prior beliefs. We proposed a six-stage consultation plan to help staff keep patients at equipoise and so facilitate trial entry and treatment acceptance[50]. Within this feasibility study we will determine if this approach is possible and successful. Secondly, UK data do not accurately identify the number of patients with HRNMIBC, what proportion of these are suitable for both RC and mBCG, and how many of these would accept randomised treatment options. Within this feasibility study we will establish accurate data about the number of eligible cases across this population and understand what proportion accept their randomised treatment allocation. We will use these findings to power the phase III comparative study. Finally, there are very few reliable data about quality of life with mBCG and none that compare this directly to RC. Within this study we will produce these data within 60 patients (30 for each arm) and so allow this endpoint to be modelled for the larger phase III study.

For peer review only

For peer review only - http://bmjopen.bmj.com/site/about/guidelines.xhtml

ETHICS AND DISSEMINATION

The study has ethical approval from Research Ethics Service Committee Yorkshire & Humber – South Yorkshire (reference 16/YH/0268). The results of the study will be published in peer-reviewed publications and will be presented at relevant national and international conferences. We will work with our patient panel of bladder cancer survivors to develop lay reports to disseminate research findings to patient groups and the clinical teams at participating sites.

Availability of data

The CTRU will control the final trial dataset and any requests for access will be reviewed by the TMG and TSC, subject to existing contractual arrangements with the funders. The protocol, sample case report forms and participant information are available on a case by case basis as agreed by the TMG, upon request to the corresponding author.

Trial Status

The trial opened to recruitment in October 2016 using protocol version 2.0 (08/08/2016) and is due to close in March 2018.

The protocol was amended to version 3 in October 2016 to account for additional inclusion and exclusion criteria, and updated surgeon accreditation criteria. The protocol was amended to version 4 in November 2016 to further update the inclusion criteria and surgeon accreditation criteria. Both amendments were reviewed and approved by the sponsor, and the National Research Ethics Service Committee Yorkshire & Humber – South Yorkshire (reference 16/YH/0268). Protocol amendments are disseminated to relevant parties by CTRU.

For peer review only - http://bmjopen.bmj.com/site/about/guidelines.xhtml

DECLARATIONS

Authors' Contributions

Conception and design of the BRAVO trial: JWFC, JBO, HP, VH, MC, JMB.

Protocol/Patient Information Sheet: JWFC, JBO, HP, MC, VH, MT, LG.

Writing of manuscript: JBO, JWFC, HP, MC, MT, KG, MJ, SJ, RC, MS, MD, PK, VH, LG, JMB.

All authors have read and approved the final manuscript. The trial will comply with the authorship criteria recommended by the International Committee of Medical Journal Editors.

Acknowledgements & funding

We gratefully acknowledge the ongoing support of participants, principal investigators, research nurses, MDT coordinators, data managers and other site staff who have been responsible for setting up, recruiting participants and collecting the data for the trial. This trial was funded by Yorkshire Cancer Research (Study S388) and we acknowledge the help of Kathryn Scott in the development of this project. The funder had no role in the design, analysis or collection of the data; in writing the manuscript; or in the decision to submit the manuscript for publication. We are grateful for the trial oversight provided by the sponsor Sheffield Teaching Hospitals NHS Trust and the members of the TSC.

Competing Interests

The other authors declare no other competing interests

For peer review only

Figure 1: Study Flow Diagram

For peer review only

For peer review only - http://bmjopen.bmj.com/site/about/guidelines.xhtml

REFERENCE LIST

1. Chavan S, Bray F, Lortet-Tieulent J, et al. International variations in bladder cancer incidence and mortality. European urology 2014;**66**(1):59-73.

2. Malmstrom PU, Sylvester RJ, Crawford DE, et al. An individual patient data meta-analysis of the long-term outcome of randomised studies comparing intravesical mitomycin C versus bacillus Calmette-Guerin for non-muscle-invasive bladder cancer. European urology 2009;**56**(2):247-56.

3. Sylvester RJ, van der Meijden AP, Oosterlinck W, et al. Predicting recurrence and progression in individual patients with stage Ta T1 bladder cancer using EORTC risk tables: a combined analysis of 2596 patients from seven EORTC trials. European urology 2006;**49**(3):466-5; discussion 75-7.

4. Kulkarni GS, Hakenberg OW, Gschwend JE, et al. An updated critical analysis of the treatment strategy for newly diagnosed high-grade T1 (previously T1G3) bladder cancer. European urology 2010;**57**(1):60-70.

5. Thomas F, Noon AP, Rubin N, et al. Comparative outcomes of primary, recurrent and progressive high-risk non-muscle invasive bladder cancer. European urology 2013;**63**(1):145-54.

6. Sylvester RJ, van der MA, Lamm DL. Intravesical bacillus Calmette-Guerin reduces the risk of progression in patients with superficial bladder cancer: a meta-analysis of the published results of randomized clinical trials. J Urol 2002;**168**(5):1964-70.

7. Mostafid AH, Palou Redorta J, Sylvester R, et al. Therapeutic options in high risk non-muscle invasive bladder cancer during the current worldwide shortage of Bacille Calmette Guerin. European urology 2015;**In Press**.

8. Lamm DL, van der Meijden PM, Morales A, et al. Incidence and treatment of complications of bacillus Calmette-Guerin intravesical therapy in superficial bladder cancer. J Urol 1992;**147**(3):596-600.

9. van der Meijden AP, Sylvester RJ, Oosterlinck W, et al. Maintenance Bacillus Calmette-Guerin for Ta T1 bladder tumors is not associated with increased toxicity: results from a European Organisation for Research and Treatment of Cancer Genito-Urinary Group Phase III Trial. European urology 2003;**44**(4):429-34.

10. Lamm DL, Blumenstein BA, Crissman JD, et al. Maintenance bacillus Calmette-Guerin immunotherapy for recurrent TA, T1 and carcinoma in situ transitional cell carcinoma of the bladder: a randomized Southwest Oncology Group Study. J Urol 2000;**163**(4):1124-9.

11. Lambert EH, Pierorazio PM, Olsson CA, et al. The increasing use of intravesical therapies for stage T1 bladder cancer coincides with decreasing survival after cystectomy. BJU Int 2007;**100**(1):33-6.

For peer review only

12. Schrier BP, Hollander MP, van Rhijn BW, et al. Prognosis of muscle-invasive bladder cancer: difference between primary and progressive tumours and implications for therapy. European urology 2004;**45**(3):292-6.

13. Huguet J, Crego M, Sabate S, et al. Cystectomy in patients with high risk superficial bladder tumors who fail intravesical BCG therapy: pre-cystectomy prostate involvement as a prognostic factor. European urology 2005;**48**(1):53-9; discussion 59.

14. Denzinger S, Fritsche HM, Otto W, et al. Early versus deferred cystectomy for initial high-risk pT1G3 urothelial carcinoma of the bladder: do risk factors define feasibility of bladder-sparing approach? European urology 2008;**53**(1):146-52.

15. Solsona E, Iborra I, Rubio J, et al. The optimum timing of radical cystectomy for patients with recurrent high-risk superficial bladder tumour. BJU Int 2004;**94**(9):1258-62.

16. Shabsigh A, Korets R, Vora KC, et al. Defining Early Morbidity of Radical Cystectomy for Patients with Bladder Cancer Using a Standardized Reporting Methodology. European urology 2009;**55**(1):164-74.

17. Hardt J, Filipas D, Hohenfellner R, et al. Quality of life in patients with bladder carcinoma after cystectomy: first results of a prospective study. Qual Life Res 2000;**9**(1):1-12.

18. Hautmann RE, Volkmer BG, Gust K. Quantification of the survival benefit of early versus deferred cystectomy in high-risk non-muscle invasive bladder cancer (T1 G3). World J Urol 2009;**27**(3):347-51.

19. Lane JA, Donovan JL, Davis M, et al. Active monitoring, radical prostatectomy, or radiotherapy for localised prostate cancer: study design and diagnostic and baseline results of the ProtecT randomised phase 3 trial. Lancet Oncol 2014;**15**(10):1109-18.

20. Stein JP, Penson DF. Invasive T1 bladder cancer: indications and rationale for radical cystectomy. BJU Int 2008;**102**(3):270-5.

21. NICE. Bladder cancer Guidelines. Secondary Bladder cancer Guidelines 2015. https://www.nice.org.uk/guidance/indevelopment/gid-cgwave0600/documents.

22. Huddart RA, Hall E, Lewis R, et al. Life and death of spare (selective bladder preservation against radical excision): reflections on why the spare trial closed. BJU Int 2010;**106**(6):753-5.

23. Paramasivan S, Huddart R, Hall E, et al. Key issues in recruitment to randomised controlled trials with very different interventions: a qualitative investigation of recruitment to the SPARE trial (CRUK/07/011). Trials 2011;**12**(78).

24. Moynihan C, Lewis R, Hall E, et al. The Patient Deficit Model Overturned: a qualitative study of patients' perceptions of invitation to participate in a randomised controlled trial comparing selective bladder preservation against surgery in muscle invasive bladder cancer (SPARE, CRUK/07/011). Trials 2012;**13**(228).

25. Kaur G, Smyth R, Williamson P. Developing a survey of barriers and facilitators to recruitment in randomised controlled trials. Trials 2012;**13**(218).

For peer review only

For peer review only - http://bmjopen.bmj.com/site/about/guidelines.xhtml

26. Fletcher B, Gheorghe A, Moore D, et al. Improving the recruitment activity of clinicians in randomised controleed trials. BMJ open 2012;**2: e000496**.

27. Mills N, Blazeby J, Hamdy F, et al. Training recruiters to randomized trials to facilitate recruitment and informed consnet by exploring patients' treatment preferences. Trials 2014;**15**(323).

28. Mills N, Donovan J, Wade J, et al. Exploring treatment preferences facilitated recruitment to randomised controlled trials. Journal of clinical epidemiology 2011;**64**:1127-36.

29. JN E, G S, JI E. *World Health Organization classification of tumours. Pathology and genetics of tumours of the urinary system and male genital organs*. Lyon, France: IARC Press, 2004.

30. *Histological typing of urinary bladder tumours*. Geneva, Switzerland: World Health Organization, 1973.

31. Boustead GB, Fowler S, Swamy R, et al. Stage, grade and pathological characteristics of bladder cancer in the UK: British Association of Urological Surgeons (BAUS) urological tumour registry. BJU Int 2014;**113**(6):924-30.

32. National Cancer Intelligence Network Cancer e-atlas. Secondary National Cancer Intelligence Network Cancer e-atlas. http://www.ncin.org.uk/cancer_information_tools/eatlas.

33. Thomas F, Rosario DJ, Rubin N, et al. The long-term outcome of treated high-risk non-muscle invasive bladder cancer: Time to change treatment paradigm? Cancer 2012;**118**(22):5525-34.

34. Konety BR, Dhawan V, Allareddy V, et al. Impact of hospital and surgeon volume on in-hospital mortality from radical cystectomy: data from the health care utilization project. J Urol 2005;**173**(5):1695-700.

35. Fearon KC, Ljungqvist O, Von Meyenfeldt M, et al. Enhanced recovery after surgery: a consensus review of clinical care for patients undergoing colonic resection. Clin Nutr 2005;**24**(3):466-77.

36. Azhar RA, Bochner B, Catto J, et al. Enhanced Recovery after Urological Surgery: A Contemporary Systematic Review of Outcomes, Key Elements, and Research Needs. European urology 2016;**70**(1):176-87.

37. Rabin R, de Charro F. EQ-5D: a measure of health status from the EuroQol Group. Ann Med 2001;**33**(5):337-43.

38. Aaronson NK, Ahmedzai S, Bergman B, et al. The European Organization for Research and Treatment of Cancer QLQ-C30: a quality-of-life instrument for use in international clinical trials in oncology. J Natl Cancer Inst 1993;**85**(5):365-76.

39. Common Terminology Criteria for Adverse Events (CTCAE) Version 4.0. http://evs.nci.nih.gov/ftp1/CTCAE/CTCAE_4.03_2010-06-14_QuickReference_5x7.pdf: U.S Department of Health and Human Services, National Institute of Health, 2009.

For peer review only - http://bmjopen.bmj.com/site/about/guidelines.xhtml

40. Dindo D, Demartines N, Clavien PA. Classification of surgical complications: a new proposal with evaluation in a cohort of 6336 patients and results of a survey. Ann Surg 2004;**240**(2):205-13.

41. Edmondson AJ, Birtwistle JC, Catto JW, et al. The patients' experience of a bladder cancer diagnosis: a systematic review of the qualitative evidence. J Cancer Surviv 2017.

42. McDonald AM, Knight RC, Campbell MK, et al. What influences recruitment to randomised controlled trials? A review of trials funded by two UK funding agencies. Trials 2006;**7**(9).

43. Kaur G, Hutchison I, H M, et al. Barriers to recruitment for surgical trials in head and neck oncology: a survey of trial investigators. BMJ open 2013.

44. Wade J, Donovan J, Lane J, et al. It's not just what you say, it's also how you say it: opening the 'black box' of informed consent appointments in randomised controlled trials. Social science & medicine 2009;**68**(11):2018-28.

45. Braun V, Clarke V. Using Thematic Analysis in Psychology. Qualitative Research in Psychology 2006;**3**:77-101.

46. Joffe H, L. Y. Content and thematic analysis. In: Marks DF, L Y, eds. Research Methods for Clinical and Health Psychology. London: SAGE Publications Ltd, 2004.

47. Glaser BG, Strauss AL. *The Discovery of Grounded Theory*. Chicago: Aldine, 1967.

48. Charmay K. *Constructing grounded theory: a practical guide through qualitative analysis*. London: Sage, 2006.

49. Donovan J, Mills N, Smith M, et al. Quality improvement report: Improving design and conduct of randomised trials by embedding them in qualitative research: ProtecT (prostate testing for cancer and treatment) study. Commentary: presenting unbiased information to patients can be difficult. Bmj 2002;**325**(7367):766-70.

50. Realpe A, Adams A, Wall P, et al. A new simple six-step model to promote recruitment to RCTs was developed and successfully implemented. Journal of clinical epidemiology 2016;**76**:166-74.

For peer review only - http://bmjopen.bmj.com/site/about/guidelines.xhtml

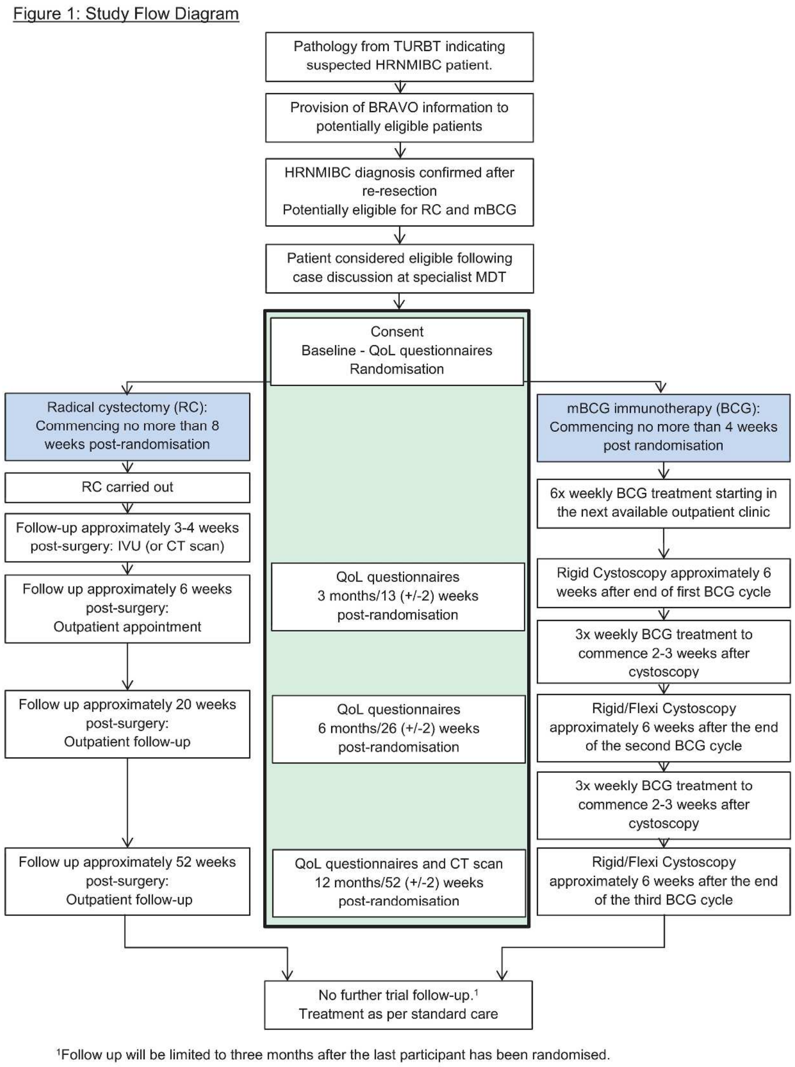

Figure 1: Study Flow Diagram

297x420mm (300 x 300 DPI)

Supplementary File 1

Qualitative Sub Study 1: Understanding patients' and health professionals' beliefs about BCG and radical cystectomy and potential barriers to recruitment.

Introduction

Lessons have been learned from previous large surgical randomised controlled trials (RCTs) (1), which suggest that when trials compare very different interventions, there are likely to be significant barriers to recruitment. A previous bladder cancer trial struggled to recruit (CRUK-SPARE trial), so this study comprises the preliminary work necessary for a feasibility RCT of mBCG versus primary radical cystectomy.

There are many barriers to recruitment and in the context of surgical trials we know that patient-factors, clinical-team factors, and information and consent related issues have all been identified as important considerations (2, 3). There will be a range of reasons for declining participation in the clinical trial including a lack of interest (4), not feeling well enough (5), fear of increased time commitments (4), and patient preferences (6). However, decisions not to participate may also be related to patients' misunderstandings regarding clinical trials (7) or how the healthcare professionals involved present the design and objectives of the study to the patient (7), and how the patient assimilates this information.

In the case of surgical trials, a need for staff training has been identified to ensure that both arms of the trial are presented in a balanced way so that patients understand the relative strengths and weaknesses of each, and there is also a recognised need for training about how to describe RCT methods (8). Radical cystectomy and BCG have been around in clinical practice for many years, so patients and health professionals may have a strong preference for either surgery or BCG and could feel that this choice is taken out of their hands by the randomization process. Understanding and addressing these issues will be crucial to the success of the feasibility trial whose aim will be to demonstrate that recruiting to a larger scale phase III trial is feasible.

It is therefore important that we have a clear understanding of patients' and health professionals' beliefs about these two treatments and ensure information presented to patients

For peer review only

by health professionals is done so in a way which minimises potential biases and facilitates an informed decision about participation. To address this, a tailored training package will be developed to enable staff to elicit and sensitively explore patient preferences for treatment, and facilitate an informed decision about participation. The development of the training package will be informed by existing evidence of what works (2, 9, 10) and content specific evidence derived from interviews with patients and healthcare staff to explore: a) treatment perceptions, b) barriers to participation, c) training needs of site staff.

Primary aims

- To understand patients' and professionals' beliefs about the two interventions and identify potential barriers to recruitment.
- To develop a training package for health professionals to aid informed decision making with patients

Secondary aims

- To elicit patients' beliefs and experiences of the two interventions (routes to diagnosis and beliefs about treatment options)
- To understand treatment burden and quality of life following treatment
- To elicit patient expectations of likely trial burden and barriers to participation
- To elicit patient recommendations for optimal recruitment and their views about randomisation
- To elicit health professional's beliefs about treatments, barriers to participation and perceived training needs

Outcome

Using the information gathered from the interviews and focus groups, and existing literature, develop a training package and associated materials and deliver the training package to staff to improve recruitment communication with patients.

For peer review only

Phase 1: ***Understanding Health Professionals views of bladder cancer treatment***

Design: Focus group study

Setting

Counselling and recruitment to the planned RCT will occur at the cancer referral centres, but patients are likely to discuss their treatment with the consultant at their local urological unit. To better understand the treatment beliefs of the health professionals (urologists, surgeons, nurses, research nurses, MDT co-ordinators and clinical nurse specialists) that patients may come in contact with, either to receive guidance on their treatment options, or to discuss the clinical trial, we approached staff from local units and referral centres. Packs were sent to the local Principal Investigator at each consenting site.

Inclusion Criteria

Staff involved in the recruitment of patients to the feasibility trial (MDT co-ordinators, surgeons, urologists, research nurses, clinical nurse specialists).

Sampling

We conducted focus groups with health professionals involved at different stages of the diagnosis pathway and trial recruitment pathway. A purposive sampling strategy was used to ensure we interviewed people involved across the diagnosis process, plus research nurses who would be involved in recruitment to the future trial. The sample included staff at local units and referral centres; nurses, (to include clincial nurse specialists and research nurses) (n=6-8), urologists and surgeons at local units and referral centres (n=6-8). We aimed to include senior and less experienced staff in each group.

Sample identification and consent process

All staff involved in the diagnosis process at each urological unit (local units and referral centres) were invited by letter to participate. An information pack (PIS, consent form, demographics form) was sent via the local Principal Investigator to their team.

For peer review only - http://bmjopen.bmj.com/site/about/guidelines.xhtml

Procedure

Two focus groups were undertaken (one each: nurses; clinicians); interviews (telephone or face to face) were offered to those who consented but could not attend the focus group. Focus groups were lead by an experienced qualitative researcher (MT) and supported by a second researcher. Written consent was taken at the beginning of the focus group. Discussions were informed by a topic guide which was informed by existing literature, (e.g. 9) clinical input and our PPI members, to include: beliefs about, and attitudes towards the interventions, barriers to recruitment, and training needs. The focus groups were audio-recorded with permission of the participants.

Data Analysis

Due to time and funding constraints, interviews were listened to and key sections transcribed for analysis. Personally identifiable data was removed or de-identified during transcription. The focus groups were analysed first, using an inductive, thematic coding approach. These were used to devise a coding frame for the interview transcripts. One researcher (JB) coded the remaining recordings, and a second researcher (MT) examined sections of data to check robustness of the themes.

Phase 2: Understanding patient views of bladder cancer treatments

Design: Semi-structured face-to-face interviews.

Inclusion criteria

- Aged 18 years or older
- Previous high grade (or grade 3) urothelial bladder cancer or non-muscle invasive tumour (diagnosed in previous 24 months – but not less than 4 months)
- Received either radical cystectomy or MBCG (or both)
- Able to provide written informed consent
- Able to converse in English (even if not first language)
- Currently or previously under the care of the urological units in Yorkshire and Humber.

For peer review only

Exclusion criteria

- Decline participation in the study
- Unable to comply with requirements of this protocol
- Unable to give informed consent

Study Setting

Participants were recruited from seven sites, to include patients treated at both local units and cancer referral centres.

Sampling

Due to the sensitive nature of bladder cancer, in-depth, semi-structured qualitative interviews were undertaken. We aimed for maximum variation in our sampling, with participants selected on the basis of socio-demographic factors (age, gender, experience of the intervention(s), geographic spread, and time since treatment). A sample of approximately 24 to 30 patients was expected.

Sample identification and consent process

Patients fitting the inclusion criteria were identified by the clinical team from clinic databases and an approach made in person, by telephone, or by post. Patients were also identified at regular clinic appointments and an information pack provided and verbal consent sought for the patient's details to be passed to the research team. At least 48 hours was given between being given the information pack and the phone call from the research team. If no response was received, a reminder letter was sent 14 days after the date of the first letter. If no response was received to the second request, no further contact was made.

When an approach was made by post, a pack containing a letter, demographics form, PIS, expression of interest form, consent form and freepost envelope was sent to the patient inviting them to participate. On return of the expression of interest (EoI) slip and demographics form, patients were contacted by the research team to discuss the study. Once consent has been received, patients were contacted to set up an appointment. For telephone interviews, a copy of the consent form was signed by the researcher and posted to the participant. For face-to-face interviews, a copy of the signed consent form was given back to the participant on the day of the interview.

For peer review only

Patients were offered more time to consider participation and a number was provided that patients could use to contact the researcher. This recruitment strategy was selected because it minimises response bias and potentially increases the methodological rigour of the research (11).

Interview procedure

In depth semi-structured interviews were conducted with participants to elicit their beliefs about the two treatment options, their route to diagnosis, and to understand treatment burden and quality of life following treatment. A key role of the study was to understand and try to address issues around clinical trial participation, so we asked about likely trial burden, barriers to participation, recommendations for optimal recruitment and views about randomisation. Interviews were expected to last 45- 60 minutes. A topic guide was developed from the existing literature and discussions with the Chief Investigator, clinicians and Patient and Public Involvement members. Interviews were conducted by an experienced qualitative researcher. Since several studies (12, 13) show that there are no major differences in the results of telephone and face-to-face interviews, participants were given the option of a telephone interview to accommodate family and professional obligations. Interviews will be audio-recorded, with the permission of the participant.

Data analysis (as Phase 1 above)

Interviews were professionally transcribed verbatim and managed using NVivo. Personally identifiable data was removed or de-identified during transcription, and pseudonyms used. The data was analysed using Framework analysis (14) by three researchers independently coding the first three transcripts using initially inductive then deductive approaches. Codes and themes were compared after the analysis of the first three transcripts. Two researchers (AE & JB) then coded the remaining transcripts, with regular meetings with MT to ensure coding remains consistent. The analysis was further refined by using a constant comparison and contrastive approach, and looking for negative cases in order to examine for similarities and differences within and between patient groups.

Phase 3: Development of Training Package

The training package was developed from the findings of the interview and focus group data, and informed by the existing literature (9, 10). Training was delivered as a face-to-face

For peer review only

workshop delivered at 3 sites and incorporated presentations and role play exercises with simulated patients (trained individuals who are regularly used in communication skills training throughout healthcare education) (15, 16). A manual was developed to accompany the training and included: detailed information about the trial and the two treatments, information on how to discuss uncertainty (of treatment options), how to describe randomisation, how to talk to patients who express a treatment preference. The aim of the training day was to allow staff to practice their communication skills in relation to the trial and receive feedback.

Results

The findings of the work are currently being written up for publication.

Ethical issues

Confidentiality

We were mindful of protecting participant confidentiality at all times. Audio recordings were stored on a secure drive and accessed only by the researcher team. After analysis the audio recordings were destroyed. Personally identifiable data was removed during transcription and pseudonyms adopted; these bear no resemblance to the patient's identity, hospital number, DOB or similar. Participants were asked to consent to direct quotes. Paper documents (e.g. consent forms, demographic questionnaires etc.) are kept in a secure office, and electronic information stored on University computers which are password protected. The file in which codes are linked to patients' names is stored on a password protected computer on a secure network. All data will be archived in accordance with University of Leeds and University of Sheffield NHS Foundation Trust procedures.

Informed consent

The patients were required to sign a consent form prior to getting involved to the sub-study. Those unable to consent for themselves were excluded from participating.

Time frame: October 2015 to September 2016.

Patient and Public Involvement

For peer review only

One lay member (PK), was involved in the development of the proposal. PK was involved in the design of the study, and has commented on the wording of this protocol, as well as the PIS, consent forms and topic guides used in this study. PK will remain involved in the study. A patient group was set up for the project and provided input into the study at key points in the project (study design, development of training manual, data analysis, and dissemination). Lay members participated in the training events to co-deliver the training package.

REC Review and reports

Approval for the study was sought and obtained (REF 15/LO/1864) and the study obtained R & D approvals from the NHS Trusts involved.

External Peer Review

This study is funded by Yorkshire Cancer Research and has undergone independent expert peer review, including review by a qualitative methodologist and a clinician.

For peer review only

References

1. Lane JA, Donovan JL, Davis M, Walsh E, Dedman D, Down L, et al. Active monitoring, radical prostatectomy, or radiotherapy for localised prostate cancer: study design and diagnostic and baseline results of the ProtecT randomised phase 3 trial. The Lancet Oncology. 2014;15(10):1109-18.
2. Moynihan C, Lewis R, Hall E, Jones E, Birtle A, Huddart R, et al. The Patient Deficit Model Overturned: a qualitative study of patients' perceptions of invitation to participate in a randomized controlled trial comparing selective bladder preservation against surgery in muscle invasive bladder cancer (SPARE, CRUK/07/011). Trials [Electronic Resource]. 2012;13:228.
3. Kaur G, Hutchinson I, Mehanna H, Williamson P, Shaw R, Tudur Smith C. Barriers to recruitment for surgical trials in head and neck oncology: a survey of trial investigators. BMJ Open [Internet]. 2013.
4. Biedrzycki BA, editor Decision making for cancer clinical trial participation: a systematic review. Oncology nursing forum; 2010.
5. NIHR-CRNCC. New from the Network [webpage]. 2011 [cited 2015]. Available from: http://www.crncc.nihr.ac.uk/Resources/NIHR%20CRN%20CC/Documents/NFTNW%20Issue%202%20Qtr%202%202010.pdf.
6. Mills EJ, Seely D, Rachlis B, Griffith L, Wu P, Wilson K, et al. Barriers to participation in clinical trials of cancer: a meta-analysis and systematic review of patient-reported factors. The Lancet Oncology. 2006;7(2):141-8.
7. Hussain-Gambles M, Leese B, Atkin K, Brown J, Mason S, Tovey P. Involving South Asian patients in clinical trials. 2004.
8. Fletcher B, Gheorghe A, Moore D, Wilson S, Damery S. Improving the recruitment activity of clinicians in randomised controlled trials: a systematic review. BMJ open. 2012;2(1):e000496.
9. Mills N, Blazeby J, Hamdy F, Neal DE, Campbell B, Wilson C, et al. Training recruiters to randomised trials to facilitate recruitment and informed consent by exploring patients' treatment preferences. Trials [Electronic Resource]. 2014;15(323).
10. Realpe A, Adams A, Wall P, Griffin D, Donovan JL. A new simple six-step model to promote recruitment to RCTs was developed and successfully implemented. Journal of clinical epidemiology. 2016;76:166-74.
11. Hewison J, Haines A. Confidentiality and consent in medical research: overcoming barriers to recruitment in health research. BMJ: British Medical Journal. 2006;333(7562):300.
12. Novick G. Is there a bias against telephone interviews in qualitative research? Research in nursing & health. 2008;31(4):391-8.
13. Sturges JE, Hanrahan KJ. Comparing telephone and face-to-face qualitative interviewing: a research note. Qualitative Research. 2004;4(1):107-18.
14. Ritchie J, Spencer L, O'Connor W. Carrying out qualitative analysis In: Ritchie J, Lewis J, editors. Qualitative Research Practice: A guide for social science students and researchers. London: Sage; 2003. p. 219-62.
15. Wykurz G, Kelly D. Developing the role of patients as teachers: a literature review. BMJ. 2002;325(818-21).
16. Bokken L, Rethans J, Scherpbier A, Van der Vieuten C. Strengths and weaknesses of simulated and real patients in the teaching of skills to medical students: a review. Simulation in Healthcare: The Journal of the Society of Medical Simulation. 2008;3:161-69.

For peer review only

STANDARD PROTOCOL ITEMS: RECOMMENDATIONS FOR INTERVENTIONAL TRIALS

SPIRIT 2013 Checklist: Recommended items to address in a clinical trial protocol and related documents*

Section/item	Item No	Description	Addressed on page number
**Administrative information**			
Title	1	Descriptive title identifying the study design, population, interventions, and, if applicable, trial acronym	1
Trial registration	2a	Trial identifier and registry name. If not yet registered, name of intended registry	4
	2b	All items from the World Health Organization Trial Registration Data Set	Yes, throughout.
Protocol version	3	Date and version identifier	25
Funding	4	Sources and types of financial, material, and other support	26
Roles and responsibilities	5a	Names, affiliations, and roles of protocol contributors	25-26
	5b	Name and contact information for the trial sponsor	22
	5c	Role of study sponsor and funders, if any, in study design; collection, management, analysis, and interpretation of data; writing of the report; and the decision to submit the report for publication, including whether they will have ultimate authority over any of these activities	26
	5d	Composition, roles, and responsibilities of the coordinating centre, steering committee, endpoint adjudication committee, data management team, and other individuals or groups overseeing the trial, if applicable (see Item 21a for data monitoring committee)	22-23

For peer review only

**Introduction**			
Background and rationale	6a	Description of research question and justification for undertaking the trial, including summary of relevant studies (published and unpublished) examining benefits and harms for each intervention	6-8
	6b	Explanation for choice of comparators	6-8
Objectives	7	Specific objectives or hypotheses	8
Trial design	8	Description of trial design including type of trial (eg, parallel group, crossover, factorial, single group), allocation ratio, and framework (eg, superiority, equivalence, noninferiority, exploratory)	9
**Methods: Participants, interventions, and outcomes**			
Study setting	9	Description of study settings (eg, community clinic, academic hospital) and list of countries where data will be collected. Reference to where list of study sites can be obtained	9, 12
Eligibility criteria	10	Inclusion and exclusion criteria for participants. If applicable, eligibility criteria for study centres and individuals who will perform the interventions (eg, surgeons, psychotherapists)	9-11, 15-16
Interventions	11a	Interventions for each group with sufficient detail to allow replication, including how and when they will be administered	14-15
	11b	Criteria for discontinuing or modifying allocated interventions for a given trial participant (eg, drug dose change in response to harms, participant request, or improving/worsening disease)	14-15
	11c	Strategies to improve adherence to intervention protocols, and any procedures for monitoring adherence (eg, drug tablet return, laboratory tests)	n/a
	11d	Relevant concomitant care and interventions that are permitted or prohibited during the trial	n/a
Outcomes	12	Primary, secondary, and other outcomes, including the specific measurement variable (eg, systolic blood pressure), analysis metric (eg, change from baseline, final value, time to event), method of aggregation (eg, median, proportion), and time point for each outcome. Explanation of the clinical relevance of chosen efficacy and harm outcomes is strongly recommended	8, 18-19
Participant timeline	13	Time schedule of enrolment, interventions (including any run-ins and washouts), assessments, and visits for participants. A schematic diagram is highly recommended (see Figure)	Flow diagram, 17

For peer review only - http://bmjopen.bmj.com/site/about/guidelines.xhtml

Sample size	14	Estimated number of participants needed to achieve study objectives and how it was determined, including clinical and statistical assumptions supporting any sample size calculations	11-12
Recruitment	15	Strategies for achieving adequate participant enrolment to reach target sample size	13-14, 19

Methods: Assignment of interventions (for controlled trials)

Allocation:			
Sequence generation	16a	Method of generating the allocation sequence (eg, computer-generated random numbers), and list of any factors for stratification. To reduce predictability of a random sequence, details of any planned restriction (eg, blocking) should be provided in a separate document that is unavailable to those who enrol participants or assign interventions	17-18
Allocation concealment mechanism	16b	Mechanism of implementing the allocation sequence (eg, central telephone; sequentially numbered, opaque, sealed envelopes), describing any steps to conceal the sequence until interventions are assigned	17-18
Implementation	16c	Who will generate the allocation sequence, who will enrol participants, and who will assign participants to interventions	9, 13
Blinding (masking)	17a	Who will be blinded after assignment to interventions (eg, trial participants, care providers, outcome assessors, data analysts), and how	9
	17b	If blinded, circumstances under which unblinding is permissible, and procedure for revealing a participant's allocated intervention during the trial	n/a

Methods: Data collection, management, and analysis

Data collection methods	18a	Plans for assessment and collection of outcome, baseline, and other trial data, including any related processes to promote data quality (eg, duplicate measurements, training of assessors) and a description of study instruments (eg, questionnaires, laboratory tests) along with their reliability and validity, if known. Reference to where data collection forms can be found, if not in the protocol	17, 25
	18b	Plans to promote participant retention and complete follow-up, including list of any outcome data to be collected for participants who discontinue or deviate from intervention protocols	16-17

For peer review only - http://bmjopen.bmj.com/site/about/guidelines.xhtml

Data management	19	Plans for data entry, coding, security, and storage, including any related processes to promote data quality (eg, double data entry; range checks for data values). Reference to where details of data management procedures can be found, if not in the protocol	17
Statistical methods	20a	Statistical methods for analysing primary and secondary outcomes. Reference to where other details of the statistical analysis plan can be found, if not in the protocol	18-19
	20b	Methods for any additional analyses (eg, subgroup and adjusted analyses)	n/a
	20c	Definition of analysis population relating to protocol non-adherence (eg, as randomised analysis), and any statistical methods to handle missing data (eg, multiple imputation)	18-19
**Methods: Monitoring**			
Data monitoring	21a	Composition of data monitoring committee (DMC); summary of its role and reporting structure; statement of whether it is independent from the sponsor and competing interests; and reference to where further details about its charter can be found, if not in the protocol. Alternatively, an explanation of why a DMC is not needed	22
	21b	Description of any interim analyses and stopping guidelines, including who will have access to these interim results and make the final decision to terminate the trial	n/a
Harms	22	Plans for collecting, assessing, reporting, and managing solicited and spontaneously reported adverse events and other unintended effects of trial interventions or trial conduct	19
Auditing	23	Frequency and procedures for auditing trial conduct, if any, and whether the process will be independent from investigators and the sponsor	23
**Ethics and dissemination**			
Research ethics approval	24	Plans for seeking research ethics committee/institutional review board (REC/IRB) approval	23
Protocol amendments	25	Plans for communicating important protocol modifications (eg, changes to eligibility criteria, outcomes, analyses) to relevant parties (eg, investigators, REC/IRBs, trial participants, trial registries, journals, regulators)	25

For peer review only - http://bmjopen.bmj.com/site/about/guidelines.xhtml

Consent or assent	26a	Who will obtain informed consent or assent from potential trial participants or authorised surrogates, and how (see Item 32)	13
	26b	Additional consent provisions for collection and use of participant data and biological specimens in ancillary studies, if applicable	n/a
Confidentiality	27	How personal information about potential and enrolled participants will be collected, shared, and maintained in order to protect confidentiality before, during, and after the trial	23
Declaration of interests	28	Financial and other competing interests for principal investigators for the overall trial and each study site	26
Access to data	29	Statement of who will have access to the final trial dataset, and disclosure of contractual agreements that limit such access for investigators	25
Ancillary and post-trial care	30	Provisions, if any, for ancillary and post-trial care, and for compensation to those who suffer harm from trial participation	23
Dissemination policy	31a	Plans for investigators and sponsor to communicate trial results to participants, healthcare professionals, the public, and other relevant groups (eg, via publication, reporting in results databases, or other data sharing arrangements), including any publication restrictions	25
	31b	Authorship eligibility guidelines and any intended use of professional writers	26
	31c	Plans, if any, for granting public access to the full protocol, participant-level dataset, and statistical code	25
**Appendices**			
Informed consent materials	32	Model consent form and other related documentation given to participants and authorised surrogates	25
Biological specimens	33	Plans for collection, laboratory evaluation, and storage of biological specimens for genetic or molecular analysis in the current trial and for future use in ancillary studies, if applicable	n/a

*It is strongly recommended that this checklist be read in conjunction with the SPIRIT 2013 Explanation & Elaboration for important clarification on the items. Amendments to the protocol should be tracked and dated. The SPIRIT checklist is copyrighted by the SPIRIT Group under the Creative Commons "Attribution-NonCommercial-NoDerivs 3.0 Unported" license.

For peer review only - http://bmjopen.bmj.com/site/about/guidelines.xhtml
