# Supplementary material for: Radical cystectomy (bladder removal) against intravesical BCG immunotherapy for high-risk non-muscle invasive bladder cancer (BRAVO): a protocol for a randomised controlled feasibility study
Source: BMJ Open. 2017 Aug 11;7(8):e017913. doi: 10.1136/bmjopen-2017-017913 (PMC5724134; doi:10.1136/bmjopen-2017-017913)
Supplement: Reviewer comments [file bmjopen-2017-017913.reviewer_comments.pdf]

PEER REVIEW HISTORY

BMJ Open publishes all reviews undertaken for accepted manuscripts. Reviewers are asked to complete a checklist review form (**http://bmjopen.bmj.com/site/about/resources/checklist.pdf**) and are provided with free text boxes to elaborate on their assessment. These free text comments are reproduced below.

ARTICLE DETAILS

**TITLE (PROVISIONAL)**	Radical Cystectomy (Bladder Removal) against intra-vesical BCG immunotherapy for high risk non-muscle invasive bladder cancer (BRAVO): Protocol for a randomised controlled feasibility study
**AUTHORS**	Oughton, Jamie; Poad, Heather; Twiddy, Maureen; Collinson, Michelle; Hiley, Victoria; Gordon, Kathryn; Johnson, Mark; Jain, Sunjay; Noon, Aidan; Chahal, Rohit; Simms, Matt; Dooldeniya, Mohantha; Koenig, Phillip; Goodwin, Louise; Brown, Julia; Catto, James

This article was not externally reviewed at BMJ Open. Protocols that have been independently assessed prior to submission to BMJ Open are usually fast-tracked to publication on the grounds that further substantial changes will not be possible. This independent assessment will usually be external, independent review for both a substantial grant award from a non-commercial or government funder and ethics approval.
